# Mechanical confinement matters: Unveiling the effect of two-photon polymerized 2.5D and 3D microarchitectures on neuronal YAP expression and neurite outgrowth

**DOI:** 10.1016/j.mtbio.2024.101325

**Published:** 2024-11-02

**Authors:** Ahmed Sharaf, Jean-Philippe Frimat, Angelo Accardo

**Affiliations:** a Department of Precision and Microsystems Engineering, Faculty of Mechanical Engineering, Delft University of Technology, Mekelweg 2, 2628 CD Delft, the Netherlands; b Department of Human Genetics, Leiden University Medical Center, 2333 ZA Leiden, the Netherlands

## Abstract

The effect of mechanical cues on cellular behaviour has been reported in multiple studies so far, and a specific aspect of interest is the role of mechanotransductive proteins in neuronal development. Among these, yes-associated protein (YAP) is responsible for multiple functions in neuronal development such as neuronal progenitor cells migration and differentiation while myocardin-related transcription factor A (MRTFA) facilitates neurite outgrowth and axonal pathfinding. Both proteins have indirectly intertwined fates via their signalling pathways. There is little literature investigating the roles of YAP and MRTFA *in vitro* concerning neurite outgrowth in mechanically confined microenvironments. Moreover, our understanding of their relationship in immature neurons cultured within engineered confined microenvironments is still lacking. In this study, we fabricated, via two-photon polymerization (2PP), 2.5D microgrooves and 3D polymeric microchannels, with a diameter range from 5 to 30 μm. We cultured SH-SY5Y cells and differentiated them into immature neuron-like cells on both 2.5D and 3D microstructures to investigate the effect of mechanical confinement on cell morphology and protein expression. In 2.5D microgrooves, both YAP and MRTFA nuclear/cytoplasmic (N/C) ratios exhibited maxima in the 10 μm grooves indicating a strong relation with mechanical-stress-inducing confinement. In 3D microchannels, both proteins’ N/C ratio exhibited minima in presence of 5 or 10 μm channels, a behaviour that was opposite to the ones observed in the 2.5D microgrooves and that indicates how the geometry and mechanical confinement of 3D microenvironments are unique compared to 2.5D ones due to focal adhesion, actin, and nuclear polarization. Further, especially in presence of 2.5D microgrooves, cells featured an inversely proportional relationship between YAP N/C ratio and the average neurite length. Finally, we also cultured human induced pluripotent stem cells (hiPSCs) and differentiated them into cortical neurons on the microstructures for up to 2 weeks. Interestingly, YAP and MRTFA N/C ratios also showed a maximum around the 10 μm 2.5D microgrooves, indicating the physiological relevance of our study. Our results elucidate the possible differences induced by 2.5D and 3D confining microenvironments in neuronal development and paves the way for understanding the intricate interplay between mechanotransductive proteins and their effect on neural cell fate within engineered cell microenvironments.

## Graphical abstract

**Figure:**
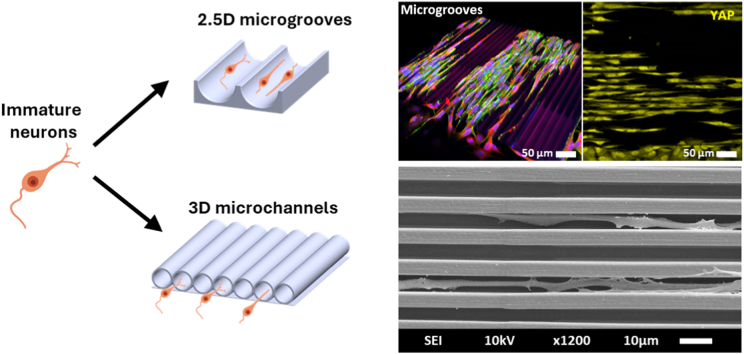

## 1 Introduction

Decision making mechanisms and gene expression within cells are affected by multiple biochemical or physical cues. Among physical cues, mechanical cues have gained a fair amount of interest and attention in the last two decades. Multiple research groups utilised a plethora of techniques for fabricating platforms with the purpose of influencing cell behaviour via mechanical cues such as stiffness, topography, and geometry [[1], [2], [3]]. A significant amount of this research focused on neurons and neuronal development [[4], [5], [6], [7], [8]]. A collection of studies over the years have shown how mechanical cues have a vital role in a range of decision making processes within neuronal development ranging from the migration and differentiation of neuronal progenitor cells to the outgrowth and guidance of neurites [7]. Mechanobiology involves the study of the effect of mechanical cues on the behaviour of cells. These effects were also observed when performing *in vitro* studies. For example, it was proven in multiple studies that neuronal and microglia guidance can be induced by patterns of micro and nanopillars [[9], [10], [11], [12]]. There are many fabrication techniques employed in neuronal mechanobiological studies such as electrospinning [13], electron beam lithography [14], microcontact printing [15] and two-photon polymerization (2PP) [16]. Among these techniques, 2PP has gained a lot of interest recently because it allows the fabrication of microstructures with complex 3D architectures and fine features with a resolution of up to 50 nm [17]. The technology of 2PP has been utilised by many groups to fabricate nanopillars for microglia phenotypic ramification [18], microcages for glioblastoma mechanobiological studies and *in vitro* treatment modelling [[19], [20], [21]], micro metamaterials for murine preosteoblasts studies [22], nano ridges for neuronal guidance of SH-SY5Y neuroblastoma [23], and microscaffolds for the development of neuronal networks from human induced pluripotent stem cells (hiPSCs) [24].

While interacting with these microstructures, cells sense mechanical cues and translate them into relevant biochemical signals via a class of proteins named mechanotransductive proteins [25]. Among these, the Yes-associated protein (YAP), which is a Hippo pathway effector, normally resides in the cytoplasm and it shuttles to the nucleus primarily when the actin filaments of the cytoskeleton are under tension [26]. Studies revealed that factors affecting the activation of YAP can be a change in the stiffness of the extracellular matrix (ECM), the area and confinement of the cell body (i.e. cell spreading), cell density, shear flow, and cell stretching [27]. Once activated, YAP interacts with transcriptional enhanced associate domain (TEAD) transcription factors in the nucleus to regulate gene expression. YAP has an evident effect on the regulation of multiple aspects of cell fate in health and disease such as their survival, self-renewal, proliferation, and differentiation [26,28]. For example, YAP activation stimulates self-renewal of human embryonic stem cells (hESCs) [29] and promotes the proliferation of vascular smooth muscle cells [30]. It has been linked also to cell necrosis when drawn towards the cytoplasm in cortical neurons in Alzheimer's patients [31]. In addition, studies have shown that YAP is responsible for cancer cell metastasis and transformation [32,33]. Moreover, YAP plays an important role in neuronal development [5]. Musah et al. showed how hESCs selectively differentiated into neurons within 10 days on soft polyacrylamide (PA) hydrogel substrates (Young's modulus (E) ∼ 0.7 kPa) even though the culture medium promoted pluripotency. This was accompanied by YAP localization in the cytoplasm and not the nucleus. In comparison, stiffer PA hydrogel substrates of E ∼10 kPa induced YAP to be located in the nucleus and was accompanied by much less pronounced neuronal differentiation [34]. Sun et al. showed similar results with compliant polydimethylsiloxane microposts' arrays (E = 3 kPa) vs. stiff ones (E = 1200 kPa) when used as substrates for hESCs for 3 days [35]. In addition to YAP, a highly relevant protein is myocardin-related transcription factor A (MRTFA/MAL/MKL1) which is a co-activator of serum response factor (SRF) in the nucleus. MRTFA acts as a sensor of the ratio of G-actin monomers to F-actin polymeric filaments [36]. In an unstimulated state, MRTFA resides in the cytoplasm and binds to G-actin monomers. When stimulated, the bond with G-actin is severed and it shuttles towards the nucleus to bind to SRF transcription factor and induces gene transcription. SRF in turn regulates the polymerization of actin in the cell. Within the nervous system, MRTFA is particularly located in the hippocampus and cerebral cortex [37]. It was shown in multiple studies that the absence of SRF has a negative effect on neurite outgrowth, axon pathfinding, and formation of neuronal circuitry [38]. Zhang et al. even illustrated that overexpression of MRTFA in the hippocampus of Alzheimer's-transfected mice reduces the accumulation of β-amyloid peptide and consequently decreases cognition defect [39]. There are multiple studies that suggest some crosstalk between the two pathways of YAP and MRTFA [[40], [41], [42]], since they are both related to actin in the cytoskeleton. Similar to YAP, MRTFA translocation from the cytoplasm to the nucleus and the subsequent activation of SRF transcription factor was shown to be manipulated by mechanical cues such as cell stretching and confinement of cellular growth volume [43,44]. Finally, within the complex of mechanotransduction, the anchor points at which the cell and ECM adhere, known as focal adhesions (FAs), play a major role. FAs are made up of multiprotein bundles and they directly and physically connect the ECM to the actin filaments of the cytoskeleton [45]. FA proteins such as vinculin and paxillin were shown to have a relationship with YAP expression and its nuclear to cytoplasmic (N/C) ratio within multiple studies [28,[46], [47], [48]].

Among mechanical cues investigated by various research groups, confinement remains an especially interesting one due to its high relevance to the mechanics of migration of neuronal progenitor cells and immature neurons within the central nervous system [7]. Although a few studies have investigated the effect of confinement on YAP N/C ratio [[47], [48], [49], [50], [51]], very little research has been performed on immature neurons in this regard. In addition, unravelling the relationship between YAP and MRTFA with respect to the elongation and outgrowth of neuronal processes at early stages of neuronal development is still at its infancy. Moreover, past studies concerned with mechanical confinement mainly used biochemical patterns of proteins such as fibronectin or 2.5D microwells made from compliant materials such as polydimethylsiloxane (PDMS). Therefore, a systematic comparison between 2.5D and 3D microenvironments has not been reported yet to the best of our knowledge. In the current study, we employed the 2PP technology to fabricate a set of 2.5D microgrooves and 3D microchannels with varying diameters (i.e. 5, 10, 20, and 30 μm) using IP-L photoresin. We then employed confocal microscopy and scanning electron microscopy (SEM) to investigate the effect of mechanical confinement on YAP and MRTFA N/C ratios as well as to identify the relationship between these ratios and the elongation of neuronal processes. Additionally, the effect of the microstructures on the early FA protein, paxillin, was studied. We cultured SH-SY5Y human neuroblastoma cells on the microstructures and differentiated them into immature neuron-like cells for 1 and 3 days. In addition, and as a proof of principle, we also cultured a more physiologically relevant hiPSC-derived neuronal model on the microgrooves and differentiated the cells for 7 and 14 days to investigate possible differences in YAP N/C ratio. Besides YAP and MRTFA, we investigated the overall expression of microtubule-associated protein 2 (MAP2) and beta-tubulin III (TUJ1) in the differentiated cells since these proteins are indicators of neuronal differentiation.

## 2 Materials and methods

### 2.1 Design of the microstructures

The design of all 2.5D and 3D microstructures employed in this study were carried out using SOLIDWORKS (Dassault Systèmes). Two sets of microstructures were designed, namely 2.5D microgrooves and 3D microchannels. Both sets had 4 different gaps/diameters 5, 10, 20, or 30 μm. From this point onward, the 2.5D microgrooves are referred to as G5, G10, G20, and G30 and the 3D microchannels will be referred to as C5, C10, C20, and C30. In the case of the 3D microchannels, we designed two additional variants featuring diameters of 5 or 10 μm and featuring a 50 μm long truncated cone attached at both ends to provide a gradual decrease of the diameter of the channels from 30 μm down to either 5 or 10 μm. This alternative design was chosen to investigate the effect of gradual confinement on the colonization of the cells in the 10 μm and, especially, 5 μm microchannels. These two 3D microchannel designs are referred to as alternative design channels (ADC) of 5 or 10 μm diameters (i.e. ADC5 and ADC10). All 2.5D microgrooves and 3D microchannels were curved and not rectangular in shape. 2.5D microgroove arrays covered an area of 750 × 750 μm^2^ and featured 15 μm high and 5 μm wide ridges. 3D microchannel arrays on the other hand covered an area of 250 × 250 μm^2^. Both structures had a base of 1 μm thick pedestals to promote adhesion to the substrate. The full detailed designs are presented in the supporting information (Figs. S1 and 2).

### 2.2 Microfabrication of the 2.5D and 3D microarchitectures

The microstructures were printed via 2PP technology by employing a Nanoscribe Photonic Professional GT+ (PPGT+) printer (Nanoscribe GmbH & Co. KG). The substrates used to perform the printing were 25 x 25 × 0.7 mm^3^ soda lime glass substrates coated with a nanometric layer of indium tin oxide (ITO) supplied by Nanoscribe GmbH & Co. KG. Prior to printing, the substrates were cleaned with acetone and isopropanol (IPA) (Sigma-Aldrich), dried, and then treated with oxygen plasma in a Diener Femto plasma cleaner for 5 min with an oxygen flowrate of 5.5 cm^3^/min at a power of 100 W and ∼0.24 mbar (40 kHz frequency). Afterwards, they were silanized with 3-(trimethoxysilyl)propyl methacrylate (MAPTMS) (TCI Chemicals) by dispensing a 30 μL droplet on a parafilm sheet and then placing the substrates face down on the droplet overnight. The silanized substrates were stored in the dark until further use. Immediately before printing, the substrates were rinsed with IPA and dried with an air gun. Afterwards, a droplet of IP-L resin was dispensed on the substrate to print the microstructures. IP-L is a proprietary acrylate-based photoresin of Nanoscribe GmbH & Co. KG and it was employed in combination with a 25x Zeiss objective (numerical aperture (NA) = 0.8) to 3D print the microstructures. This combination enabled the printing of large areas of microstructures required for biological studies with fine features (i.e. 5 μm diameter channels). The printing took place in Dip-in Laser Lithography (DiLL) mode and galvanometric mirrors-assisted scanning configuration. Printing via 2PP with the Nanoscribe PPGT + takes place in a line-by-line fashion in the horizontal direction followed by cumulative printing of consecutive layers on top of each other. The hatching distance of the horizontal lines and the slicing distance of the vertical layers were chosen to be 0.2 and 0.7 μm respectively. The direction of the hatch lines was parallel to the walls of the microstructures to prevent the effect of any competing mechanical neuronal guidance cues that maybe created if their direction was perpendicular to the walls. The laser power was set to 20 mW (equivalent to a light intensity of 0.4 TW/cm^2^ [52]) and the scanning speed was 90 mm/s. After printing, the microstructures were developed in propylene glycol monomethyl ether acetate (PGMEA) (Sigma-Aldrich) for 30 min then transferred to a second bath of clean PGMEA for 2 h. Afterwards, they were placed in an IPA bath for 5 min before placing them in NOVEC™ 7100 (Mavom BV) for 30 s to prevent structural deformation caused by capillary effect during drying. The samples were finally blow dried with an air gun.

### 2.3 Post treatment of the structures

After development, the microstructures were activated using oxygen plasma for 1 min (same parameters used in section 2.2). Immediately afterwards, they were coated with (3-Aminopropyl) triethoxysilane (APTES) (Sigma-Aldrich) by placing them in a bath of 3 % APTES in ethanol (EtOH) (Sigma-Aldrich) overnight followed by cleaning in a deionized (DI) water bath for 1 h. Coating with APTES was performed to increase the hydrophilicity of the microstructures thereby facilitating the delivery of nutrients to the cells. Amine terminated silanization was also proven to improve cell attachment and viability on 2PP acrylate-based photoresins [53]. Due to the high autofluorescence of IP-L, especially in the blue and green regions of the spectrum and since the samples were planned to be inspected via confocal microscopy after the culture of the cells, we applied a protocol to supress this autofluorescence previously developed by our group [54]. Briefly, the samples were bleached by a UV point source (Bluepoint 4 Ecocure Honle UV technology) with a wavelength range of 300–600 nm and a maximum of 375 nm for 30 min at a power of 100 % (i.e. 10,000 mW/cm^2^). The UV lamp was placed at a 1 cm distance from the samples.

The Young's modulus of the bleached microstructures was measured by compression testing with FEMTOTOOLS nanomechanical testing system (FT-NMT03) by utilising a 50 × 50 μm^2^ Si probe with a flat punch head and a measurable force range of 200,000 ± 0.5 μN. Pedestals of 30 x 30 × 20 μm^3^ (length x width x height) were used for these measurements.

### 2.4 Cell culture

#### 2.4.1 SH-SY5Y human neuroblastomas-derived immature neuron-like cells

Prior to seeding on the samples, human neuroblastoma cell line SH-SY5Y cells (Sigma-Aldrich, #94030304) were cultured in Dulbecco's Modified Eagle's Medium (DMEM)/F-12 media (1:1) (Thermo Fisher Scientific, #10565018) in presence of 10 % fetal bovine serum (FBS, Sigma-Aldrich F7524) and 1 % penicillin/streptomycin in an incubator at 37 °C and 5 % CO_2_. Once the cells were confluent, they were harvested by the use of trypsin (x1) before centrifuging them at 900 rpm for 5 min. They were then seeded on the structures at a density of 10,000 cells/cm^2^ before differentiating them into immature neuron-like cells by adding 10 μM of retinoic acid to the culture medium (Sigma-Aldrich, R2625) for 1 and 3 days.

#### 2.4.2 hiPSC-derived immature neurons

hiPSC (LUMCi003-A) [77] were thawed, resuspended in complete STEMdiff™ Neural Progenitor Media (Stem Cell Technologies, #05833) and plated on 0.1 % matrigel coated 6 well plates in complete STEMdiff™ Neural Progenitor Media. Cells were harvested for differentiation when nearly confluent by visual inspection (80 %), typically on day 7, with media refreshing every 2–3 days.

hiPSC-derived cortical neurons were generated using the STEMdiff™ midbrain neuron differentiation kit (stem cell technologies cat. No. #100-0038). Briefly, the cells were harvested using accutase (Stem Cell Technologies, #07920) and centrifuged at 400 rpm for 5 min prior to reseeding for differentiation on the 2PP samples. Cells were resuspended in full STEMdiff™ midbrain neuron differentiation media and seeded on poly-L-ornithine (100 μg/mL) (Sigma Aldrich P3655)/laminin (100 μg/mL) (Sigma Aldrich L2020) coated 2PP samples at 75,000 cells/cm^2^ in full STEMdiff™ midbrain neuron differentiation media for 7 days with daily media refreshing. After 7 days, cortical neurons were generated and half of the 2PP samples were fixed for the first time point. The remainder of the samples were further matured 1 week using the BrainPhys™ hPSC Neuron kit media (Stem Cell Technologies, #05795) with half media change every 3 days until day 14, after which samples were fixed for the second time point.

### 2.5 Immunocytochemistry

All samples were fixed in 4 % paraformaldehyde (PFA) for 15 min at room temperature (RT) and then permeabilized using 0.1 % Triton X- 100 in Phosphate Buffer Saline (PBS) solution. Blocking of the cells was done using 1 % bovine serum albumin (BSA) in PBS for 30 min at RT. Mouse anti-YAP primary antibody (Santa Cruz, sc-101199, 1:200) was then applied for 2 h in PBS containing 1 % BSA at RT. For MRTFA, we used mouse anti-MRTFA primary antibody (Santa Cruz, sc-398675, 1:200) also for 2 h in PBS containing 1 % BSA at RT. Immature neurons were stained against anti-beta-Tubulin III (TUJ-1, Sigma-Aldrich, T2200; 1:100 in 1 % BSA) and anti-MAP2 (Sigma-Aldrich, M9942; 1:200 in 1 % BSA) to study their growing processes. To visualize focal adhesions, anti-paxillin primary antibodies (Sigma-Aldrich, SAB4502553, 1:200 in 1 % BSA) were incubated for 2 h at RT. The secondary antibody (Sigma-Aldrich, SAB3700937-*Anti*- Rabbit IgG (H + L)-Texas Red® antibody, 1:500 in 1 % BSA) was used to stain paxillin and TUJ-1 antibodies by incubation for 1.5 h at RT while visualization of YAP, MRTFA, and MAP2 took place via staining also for 1.5 h at RT with the secondary antibody (Thermo Fisher Scientific, A-21235-Goat anti-Mouse IgG (H + L) Cross-Adsorbed Secondary Antibody, Alexa Fluor™ 647). As for visualizing the cytoskeleton, we used phalloidin (Thermo Fisher Scientific, ActinGreen 488 ReadyProbes, R37110) to stain F-actin. We then used NucBlue™ (Thermo Fisher Scientific, Live ReadyProbes™ Reagent Hoechst 3334, R37605) as a stain for nuclei. For both actin and Hoechst, 2 drops per mL in PBS were added and incubated for 25 min. Samples were washed 3 times with PBS afterwards and stored in PBS in the dark until imaging. All samples were stored at 4 °C in the dark until further use.

### 2.6 Confocal microscopy image acquisition

A spinning disk Dragonfly 200 High Speed Confocal microscope (Oxford Instruments Andor Ltd) was utilised to acquire all confocal images of the samples. We used a Nikon Apo LWD 25x Water Dipping objective (NA = 1.10) to acquire images of YAP, MRTFA, and TUJ-1. Imaging of these samples was carried out in PBS. A Nikon Apo total internal reflection fluorescence (TIRF) 60x oil immersion objective (NA = 1.49) was used for paxillin and MAP2 images in order to provide a higher feature resolution for the visualization of paxillin. These samples were mounted by using a ProLong™ Gold Antifade Mountant with DNA Stain DAPI (Thermo Fisher Scientific, P36941). An acquisition resolution of 1024 x 1024 px^2^ was chosen for YAP and MRTFA images of SH-SY5Y-derived immature neuron-like cells in 2.5D microgrooves while for hiPSC-derived immature neurons, we used a resolution of 2048 x 2048 px^2^. For all paxillin and 3D microchannel images, a 2048 x 2048 px^2^ resolution was chosen. Laser sources with excitation wavelengths equal to 405, 488, 561, 640 nm were employed during the acquisition. The employed emission filters were 445/46 (Hoechst), 521/38 (Actin), 594/43 (TUJ-1 and paxillin), 685/47 nm (YAP, MRTFA, MAP2). Z-stacks were acquired at 0.5 μm step size.

After confocal microscopy acquisition, each 2.5D microgroove array (750 × 750 μm^2^) was split into four z-stacks since the imaging was carried out via stitching of each array. As for the 3D microchannels, each z-stack of images included one array of 3D microchannels (250 x 250 μm^2^). The z-stacks were imported into Fiji [55] and cut into areas (along the x-y direction) that only contained the required microstructures with the cells therein. Care was taken to discard z-slices that contained any cells residing on top of the grooves or channels and not inside. Maximum z-projection images were then created from each z-stack. To identify the nuclei, the machine learning plugin Cellpose 2.0 was utilised [56]. In the case of the 3D microchannels, the plugin required some training runs to be able to identify nuclei. Once the images of the nuclei were generated and saved, they were imported in CellProfiler 4.2.6 [57] along with the relevant maximum z-projection images. Multiple pipelines were developed in CellProfiler to identify cell body, neurite length, FAs (i.e. paxillin), and MAP2 intensity per image. YAP N/C ratio was calculated by measuring the mean intensity of YAP in the nucleus and the cell body (minus the area of the nucleus) and dividing the former by the latter to obtain the result. MRTFA N/C ratio was calculated similarly. Visualizations of images throughout the manuscript were performed by using Imaris Viewer software (Oxford Instruments).

### 2.7 SEM imaging

Samples were dehydrated in order to prepare them for SEM imaging [18]. Briefly, following fixation, samples were placed in a bath of DI water 2 times for 5 min each. They were then placed in 50 % EtOH in DI water followed by a bath in 70 % and then 96 % EtOH for 15 min each. For further dehydration, hexamethyldisilazane (HMDS) (Sigma Aldrich) was used in consecutive baths of 2:1, 1:1, and 1:2 (96 % EtOH:HMDS) for 15 min each followed by 15 min of a 100 % HMDS bath for 2 times. HMDS was used due to its low surface tension to preserve the membranes and processes of the cells [[58], [59], [60]]. Finally, the samples were left to air dry before imaging. Before imaging, dehydrated samples were sputter coated with a nanometric layer of gold (≈20 nm) using a JEOL JFC-1300 auto-fine sputter coater. Imaging and further morphological characterization was carried out via a JEOL JSM-6010LA SEM (JEOL (Europe) B. V.) in high vacuum at a voltage of 10 kV.

### 2.8 Statistical analysis

For calculating the cell colonization, cell alignment, nuclear minimum and maximum Feret diameters, cell area, and neurite length of SH-SY5Y-derived immature neuron-like cells, 6 samples were employed for each time point. For YAP and MRTFA N/C ratio and MAP2 intensity, 3 samples were used for each time point. For focal adhesion number, area, and alignment, 3 samples were used for each time point. It should be noted that the 2.5D microgrooves and 3D microchannels were studied in two separate cultures. On the other hand, for the hiPSC-derived immature cortical neurons, 4 samples were used to calculate cell colonization, cell alignment, nuclear minimum and maximum Feret diameters, cell area, and neurite length. For YAP N/C ratio, 3 samples were used for each time point while for and MRTFA N/C ratio and MAP2 intensity focal adhesions number, area, and diameter, 1 sample was used for each time point. Further details of the analysis is shown in Figs. S3 and 4 in the supporting information. All data extracted from CellProfiler 4.2.6 were exported in Excel files while the analysis and presentation was performed by using an inhouse developed MATLAB (MathWorks®) code. All graphs in this study represent the mean with the standard deviation indicated therein. P-values were obtained by using two-tailed Student's t-test.

## 3 Results and discussion

### 3.1 Morphological characterization of 2.5D and 3D microfabricated structures

**Fig. 1:**
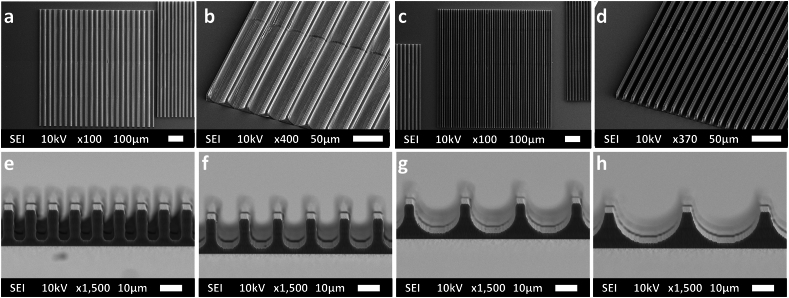
Representative SEM images of (a) and (b) G30 arrays, (c) and (d) G10 arrays. SEM images taken at 90° tilt angle of (e) G5 arrays, (f) G10 arrays, (g) G20 arrays, and (h) G30 arrays. The dimensions of the arrays of microgrooves were 750 × 750 × 15 μm^3^.

2.5D microgrooves and 3D microchannels were printed via 2PP to investigate the effect of mechanical confinement in 2.5D and 3D microenvironments on the YAP N/C ratio. Both, the 2.5D microgrooves and 3D microchannels, had diameters in the range of 5–30 μm. This specific range was chosen to be in the same range of and slightly smaller than the size of cell body and nucleus especially since there are studies suggesting that the squeezing of the nucleus is the limiting factor when attempting to mechanically confine a cell [61,62]. All 2.5D microgrooves were fabricated with high reproducibility (Fig. 1) and structural stability. No detachment was observed from the substrates even after an extended period in culture media due to their strong adhesion to the substrates. In all structures, the hatch and slice lines could be observed and helped cell adhesion, since it is known how cells preferentially grow on rough substrates [63], as well as the alignment of their processes. The 2.5D microgrooves were printed with minimal shrinkage.

**Fig. 2:**
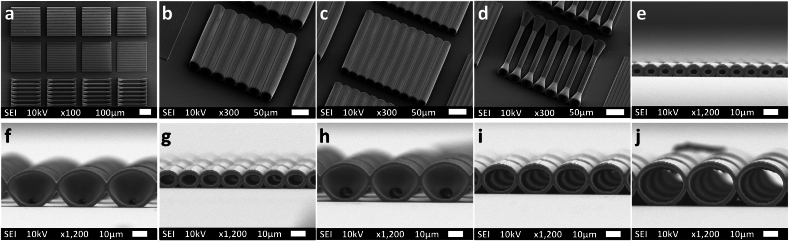
Representative SEM images of (a) 250 × 250 μm^2^ arrays of 3D microchannels, (b) C30 microchannels, (c) C20 microchannels, (d) ADC10 microchannels. SEM images taken at 90° angle of (e) C5 microchannels (f) ADC5 microchannels (g) C10 microchannels (h) ADC10 microchannels (i) C20 microchannels and (j) C30 microchannels.

As for the 3D microchannels (Fig. 2), each array of 3D microchannels was 250 × 250 μm^2^ and for each diameter of the microstructures, there were 4 arrays printed and placed next to each other longitudinally at a distance of 50 μm (Fig. 2a). An average shrinkage of 26 % was noticed along the vertical axis (i.e. z-direction) of the microstructures. The shrinkage was at a minimum of 20 % in the C30 microchannels (Fig. 2j) and increased to reach 38 % for the C5 channels (Fig. 2e). The measured dimensions of the C5 microchannels were 5.4 μm in the x-y direction and 3.3 μm in the z-direction. Shrinkage after development is considered normal due to the diffusion of the residual monomers and oligomers from the solidified resin to the solvent. A negligible amount of C5 and C10 channels still had residues from the printing process after the chemical development, thus indicating that at these diameters there was apparent residual polymerization or the possible need for a longer time of PGMEA treatment. The Young's modulus of the bleached microstructures was found to be ∼3 GPa.

### 3.2 The effect of confinement in 2.5D structures on differentiated SH-SY5Y neuroblastoma cells

The human neuroblastoma cell line SH-SY5Y was cultured on the microgrooves and differentiated into early stage neuron-like cells for 1 day (D1) and 3 days (D3) of culture in medium containing 10 μM of retinoic acid. This experiment was performed to investigate the effect of confinement in 2.5D microgrooves on the expression of YAP, the outgrowth of neurites, and the behaviour of FA molecules identified by paxillin. In addition, the relationship between YAP, MRTFA, and MAP2 expression was also investigated.

**Fig. 3:**
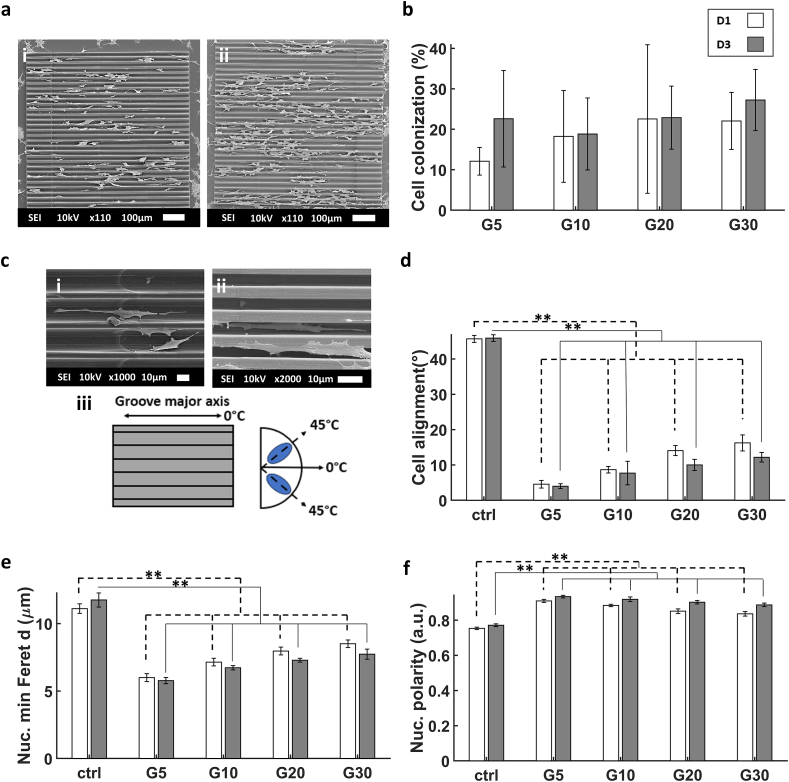
(a) Representative SEM images of (i) SH-SY5Y cells at D1 in G20, (ii) cells at D3 in G20. (b) The percentage of SH-SY5Y cell colonization in the 2.5D microgrooves calculated as the ratio between the total area of cell bodies with respect to the total area of the microgrooves. (c) SEM images showing the alignment of SH-SY5Y cells at D1 in (i) G20, and D3 in (ii) G5. (c.iii) Illustration of the major axis of the microgroove which represents the 0° angle. It also shows a schematic representation of nuclei at an angle of 45 °C with respect to the major axis of the microgrooves. (d) Degree of SH-SY5Y cell alignment on the microgrooves. (e) Average minimum Feret diameter of the cells nuclei. (f) Nuclear polarity of the SH-SY5Y cells (where 0 signifies a perfect circle and 1 a straight line). The white and grey bars represent D1 and D3 respectively. ∗ corresponds to a p-value <0.05 and ∗∗ corresponds to a p-value <0.01. P-values were obtained by two-tailed student's t-test. n = 6 samples for each timepoint.

All structures were colonized by cells, indicating good cell biocompatibility of all the microfabricated microstructures (Fig. 3a). With an initial seeding concentration of 10,000 cells/cm^2^, the colonization percentage (calculated as the ratio between the total area of cell bodies with respect to the total area of the microgrooves) ranged from 12 to 20 % for D1 of differentiation and increased in D3 to reach a range of 17–25 % (Fig. 3b). The cells migrated inside the grooves and some of them were suspended on top between two ridges (Fig. 3ci,ii). Cells exhibited also a high degree of alignment to the microgrooves compared to the cells grown on the flat ITO coated glass substrate (ctrl). There was a clear trend of alignment of the cells to the direction of the major axis of the microgrooves. This was observed by calculating the difference in the average angle of the major axis of the nuclei with respect to the major axis of the 2.5D microgrooves (Fig. 3c iii). There was a clear interaction between the processes of the cells and the hatch lines of the microstructures which likely aided the alignment of the cells. Cells on D3 showed a higher degree of alignment than D1 which could be due to the continued interaction between cells and microstructures with time or the higher degree of maturation of the cells (Fig. 3d). Similar to cell alignment, the minimum Feret diameter [64] of the nucleus showed increasing trends from G5 to G30 (Fig. 3e). The elongation of the nuclei increased with time of differentiation, as is illustrated by the simultaneous increase of nuclear polarity (Fig. 3f). Fig. S10 in the supporting information depicts 3D renders of cells in the microchannels.

**Fig. 4:**
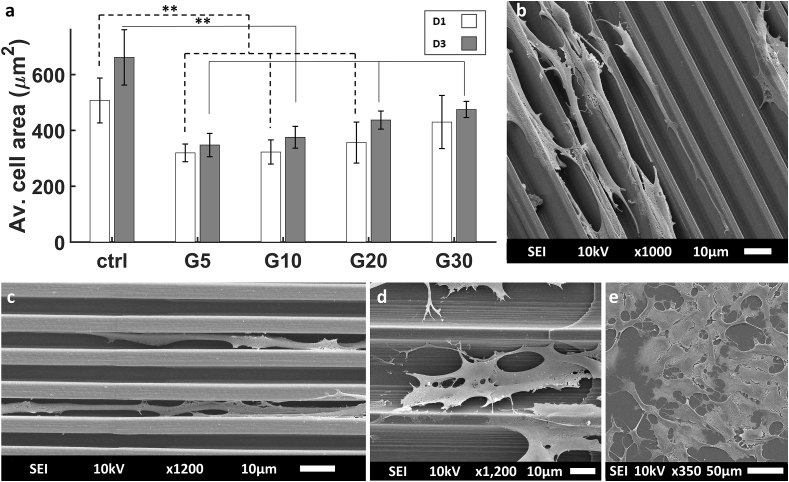
(a) Average area of the SH-SY5Y cell body (calculated from actin staining of the cytoskeleton). SEM images of SH-SY5Y immature neuron-like cells at D3 in (b) G10, (c) G5, (d) G30, and (e) on the flat ITO-coated glass substrate (ctrl). The white and grey bars represent D1 and D3 respectively. ∗ corresponds to a p-value <0.05 and ∗∗ corresponds to a p-value <0.01. P-values were obtained by two-tailed student's t-test. n = 6 samples for each timepoint.

The average area of the cell body (i.e. cytoskeleton) (Fig. 4a) showed an increasing trend from G5 to G30 indicating the ability of the nuclei and cells to stretch and fit in 2.5D microgrooves as small as 5 μm. SEM imaging showed a clear difference in terms of cell morphology and area as the cell bodies were elongated and small in G5 and G10 (Fig. 4b and c) while they were spread out and much larger in G20 and G30 (Fig. 4d). Cells cultured on the flat control showed, as expected, the largest degree of spreading and consequently the largest area (Fig. 4e).

**Fig. 5:**
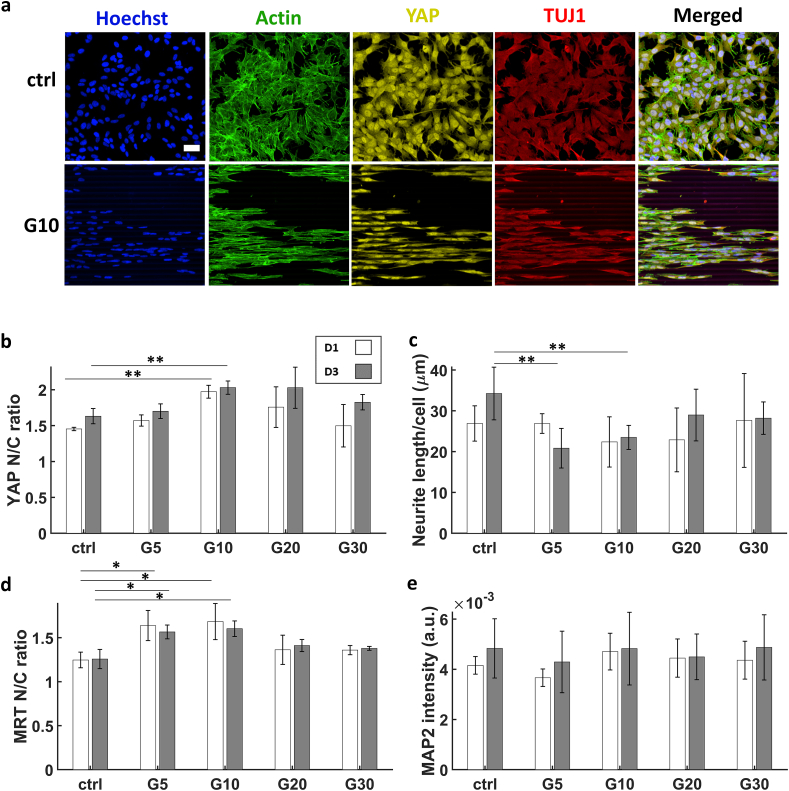
(a) Maximum Z-projection images obtained via confocal microscopy of the SH-SY5Y immature neuron-like cells at D3 of differentiation on ctrl and G10. (b) YAP N/C ratio. (c) Average total length of processes per cell. (d) MRTFA N/C ratio (e) MAP2 average intensity. The white and grey bars represent D1 and D3 respectively. ∗ corresponds to a p-value <0.05 and ∗∗ corresponds to a p-value <0.01. P-values were obtained by two-tailed student's t-test. For YAP and MRTFA N/C ratios and MAP2 intensity, n = 3 samples for each time point. For neurite average length, n = 6 samples for each time point. Scale bar = 50 μm.

Expression of YAP was evident for the ctrl and all cells in the microgrooves for both time points (Fig. 5a), which may be due to the relatively high Young's modulus of bleached IP-L (E ∼ 3 GPa) compared to the Young's modulus of brain tissue (E = 0.1–1 kPa) [65] as YAP has been shown to be concentrated in the nucleus in presence of substrates with relatively high Young's moduli in the range of tens of kPa [27,28,45,47]. Overall, the YAP N/C ratios were always greater than 1. The ratio increased from D1 to D3 for all conditions. YAP N/C ratio reached a maximum of 2 in the G10 microgrooves followed closely by G20 while the minimum (1.6) was on the flat ITO-coated glass ctrl (Fig. 5b). The trend for the average total neurite length was somewhat opposite to that of YAP since the maximum length was 34 μm on the ctrl and the minimum (20 μm) was in G5 followed by a value of 22 μm in G10 (Fig. 5c). The trend of YAP N/C ratio in relation to confined spaces goes against some literature using human mesenchymal stem cells (hMSCs) that suggest that the smaller the area of the cell body, the lower the YAP N/C ratio [47,48]. However, other studies employing mouse embryonic stem cells (mESCs) have shown that upon culturing single mESCs in 35 x 35 vs. 15 x 15 μm^2^ wells made from PDMS, the YAP N/C ratio increased in the smaller wells [66] while the study of Wada et al. also reported a threshold of YAP N/C ratio based on cell area when using NIH3T3 cells [49]. Our results show that within 2.5D microgrooves, there is a threshold of increase of the YAP N/C ratio around the 10–20 μm diameter microgrooves. The difference between our results and those mentioned in literature may be caused by a number of reasons. First, the type of cells used in the current study (SH-SY5Y human neuroblastomas) are different from those in the other mentioned studies. Second, the material used in the current study is different. The Young's modulus of bleached IP-L is 3 GPa while the one of PDMS is in the range of MPa. Third, in the studies conducted by Dupont et al. and Nardone et al., the confinement was applied by printing patterns of fibronectin squares to confine single cells [47,48]. On the other hand, in the current study and in the one of Bertels et al. [66], confinement was applied mechanically via culturing the cells in 2.5D microgrooves that are designed to contain them in small spaces comparable to that of the diameter of the nuclei. This difference in chemical vs. mechanical confinement was also observed by Rianna et al. when culturing U2OS cells in PDMS microchannels vs. fibronectin patterned lines of the same width (5 μm) [67]. Fourth, in the other studies, cells were confined in square-like wells (i.e. isotropic confinement) while in our study, the confinement took place in long aspect ratio (length/diameter) microgrooves which resulted in a highly polar configuration of the nuclei, the bodies of the cells, and the FAs (i.e. anisotropic confinement). This may suggest that isotropy also plays a role in the expression and behaviour of YAP N/C ratio. This observation is corroborated by other studies such as that of Li et al. in which they find a threshold of YAP N/C ratio of Dental pulp stem cells (DPSCs) that directly relates to the aspect ratio of the cells [51]. Finally, all mentioned studies focused on single cells while in the current study, cell-cell interaction is present, fostering network formation. In addition, YAP N/C ratio has been shown to be negatively affected by high density of cells [27,28].

As for the MRTFA N/C ratio, the maximum (1.6) was also at G10 and minimum (1.3) at the ctrl although it was different from YAP in the fact that the second largest ratio was at G5 (Fig. 5d and Fig. S5 in the supporting information). This proves how mechanical confinement can indeed affect the behaviour of MRTFA. A possible explanation of our results is that when the cells are under a relatively large amount of mechanical stress that is induced by confinement and/or high polarization (like in G5 and G10), MRTFA is mostly located in the nucleus since the polymerization of G-actin monomers into F-actin filaments increases significantly. The mean intensity of MAP2 showed a maximum at G30 followed by G10 with a minimum at G5. MAP2 intensity slightly increased from D1 to D3 for all conditions. However, the effect of confinement in the grooves on the expression of MAP2 was not statistically significant (Fig. 5e).

**Fig. 6:**
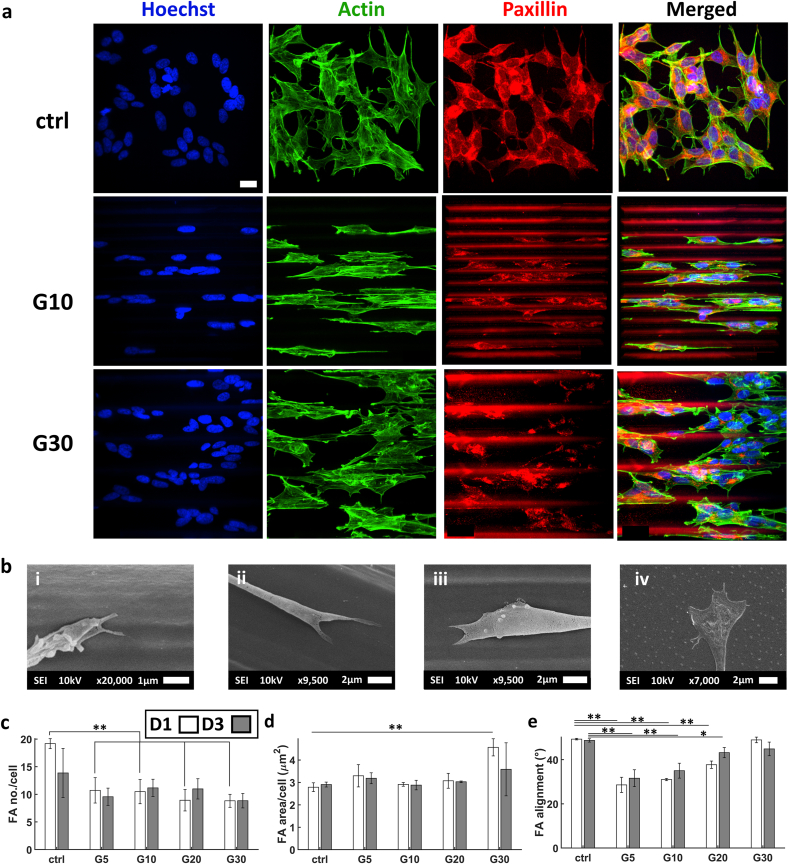
(a) Confocal maximum Z-projection of SH-SY5Y immature neuron-like cells at D3. (b) SEM images of filopodia adhering to 2.5D microgrooves (i), (ii), (iii) and the flat ITO-coated glass ctrl (iv). (c) The average number of FAs per cell. (d) Average FA area per cell. (e) Average alignment of FAs. The white and grey bars represent D1 and D3 respectively. ∗ corresponds to a p-value <0.05 and ∗∗ corresponds to a p-value <0.01. P-values were obtained by two-tailed student's t-test. n = 3 samples for each time point. Scale bar = 20 μm.

Similar to the behaviour of the nuclei, FAs exhibited a great deal of alignment within the grooves compared to the ctrl as shown in images acquired via confocal microscopy (Fig. 6a). The interaction between the filopodia of the growth cone (where most of the FAs are concentrated) and the hatch lines of the structures was evident when qualitatively investigating via SEM images (Fig. 6bi-iii). This was contrary to the spread out filopodia exhibited on the flat ctrl (Fig. 6biv). As aforementioned, this interaction increased the alignment since the hatch line distances were of the same scale as that of the filopodia (100–300 nm in size) [68]. On the other hand, such directed growth was not noticed on the flat ctrl surface since the filopodia were widely spread across the substrate.

The number of FAs per cell showed a significant difference between the cells cultured on the flat ctrl and those in any of the microgrooves with a maximum at G10 and a minimum at G30 (Fig. 6c). This may be linked to the higher YAP N/C ratio since YAP was shown to be affected by the behaviour of FAs [46]. The average area of FAs per cell did not show a specific trend since it was comparable for all cases except for G30 where there was a maximum (Fig. 6d) at D1. As for their alignment along the major axis of the 2.5D microgrooves, FAs exhibited a clear trend where the highest alignment was in G5 (Fig. 6e).

### 3.3 The effect of confinement in 3D microchannels on differentiated SH-SY5Y neuroblastoma cells

In order to assess the effect of fully confining 3D microenvironments, 3D microchannels of diameters ranging from 5 to 30 μm were printed via 2PP and, similar to the 2.5D microgrooves, SH-SY5Y cells were cultured and differentiated into immature neuron-like cells for 1 and 3 days. In order to determine the optimal length of the 3D microchannels, we performed an experiment in which SH-SY5Y cells were cultured on C30 microchannels and allowed to differentiate into neuron-like cells for 1 day. We tested channels of 250, 500, and 750 μm length. Our results showed that the 250 μm channels fostered the largest degree of cell colonization (Supporting information Fig. S8). The reason for this behaviour can be attributed to the fact that the length of the longer channels hampered the diffusion of nutrients to the cells growing therein. It is also possible that the shorter channels fostered inter-cellular communication, thus having a direct influence on cell colonization.

**Fig. 7:**
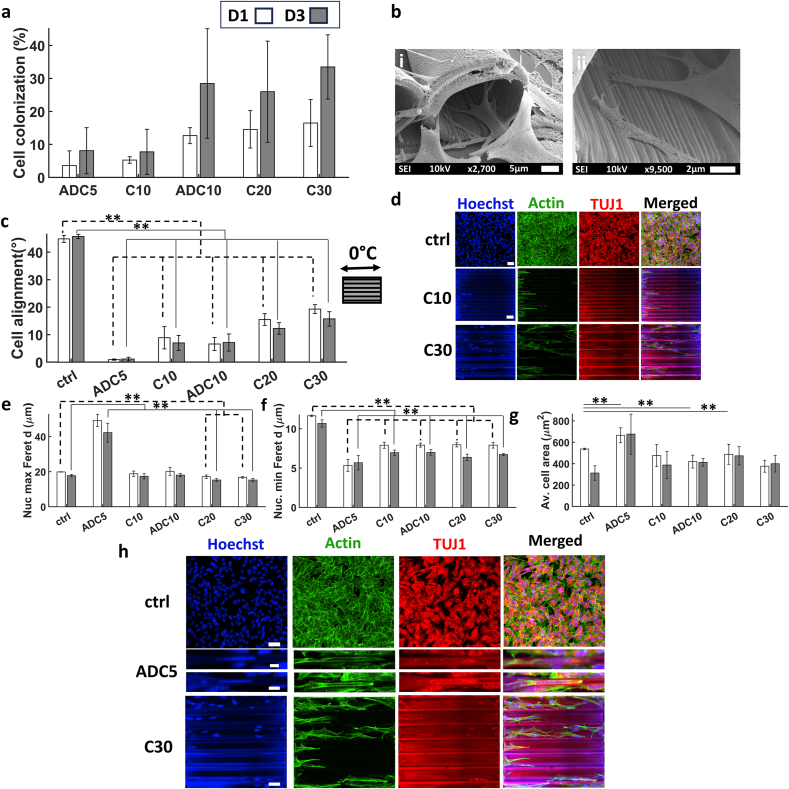
(a) The percentage of SH-SY5Y cell colonization of in the 3D microchannels calculated as the ratio between the total area of cell bodies with respect to the total area of the 3D microchannels. (b) Representative SEM images of (i) and (ii) cells probing the entrance of the 3D microchannels via filopodia. (c) Degree of cell alignment on the microchannels. (d) Confocal maximum Z-projection images of the inner volume of the microchannels showing the alignment of cells at D3. Scale bar in the first row = 50 μm and the second row = 30 μm. (e) The average maximum Feret diameter of the cells. (f) The average minimum Feret diameter of the cells. (g) The average cell body area. (h) Confocal maximum Z-projection images of the inner volume of the channels exhibiting the substantial elongation of the cell nuclei in ADC5 compared to other channels and the flat substrate. The white and grey bars represent D1 and D3 respectively. Scale bars in the first, second, third, and fourth rows = 50, 10, 15 and 30 μm respectively. ∗ corresponds to a p-value <0.05 and ∗∗ corresponds to a p-value <0.01. P-values were obtained by two-tailed student's t-test. n = 6 samples for each time point.

An investigation of cell colonization showed that all structures were colonized by cells with the exception of C5 microchannels. Interestingly, the modified design of ADC5 and ADC10 improved the colonization of cells by 8 and 20 % compared to the C5 and C10 designs respectively (Fig. 7a). Qualitative assessment via SEM imaging illustrated how cells probed the entrance of the 3D microchannels using their filopodia (Fig. 7b). The images show that the hatch line distances fostered the interaction between the filopodia and the microstructures thereby possibly increasing the chances of the cells colonizing the microchannels. Cell alignment showed the expected trend where the highest degree of alignment was in ADC5 and it decreased gradually until C30 (Fig. 7c and d). One particular observation was the significant elongation of nuclei in the ADC5 channels as shown by the maximum Feret diameter which reached 42 μm at D3 (Fig. 7e). This was accompanied by a decrease in the minimum Feret diameter to 5 μm (Fig. 7f). This phenomenon was also reflected in the area of the nuclei (Supporting information Fig. S9) and the average cell area where the maximum was at ADC5 as well (Fig. 7g and h). Fig. S11 in the supporting information depicts 3D renders of cells in the microchannels.

**Fig. 8:**
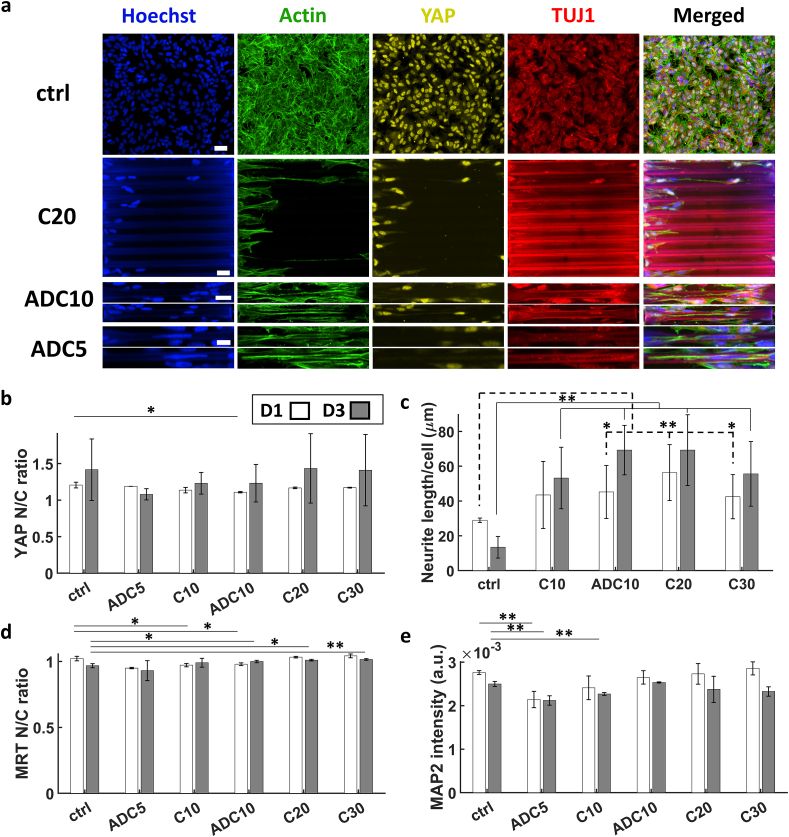
(a) Maximum Z-projection images obtained via confocal microscopy of the SH-SY5Y immature neuron-like cells at D3 of differentiation. Scale bars in the first, second, third, and fifth rows = 50, 30, 30 and 15 μm respectively. (b) YAP N/C ratio. (c) Average total length of processes per cell. (d) MRTFA N/C ratio. (e) MAP2 average intensity. The white and grey bars represent D1 and D3 respectively. ∗ corresponds to a p-value <0.05 and ∗∗ corresponds to a p-value <0.01. P-values were obtained by two-tailed student's t-test. For YAP and MRTFA N/C ratios and MAP2 intensity, n = 3 samples for each time point. For neurite average length, n = 6 samples for each time point.

The ratio of YAP N/C was similar to that of the 2.5D microgrooves in the sense that it was larger than 1 for all cases and the ratios in D3 were larger than those in D1. We noticed that some cells in C10 and ADC10 showed very little expression of YAP altogether while most cells in ADC5 showed almost no expression of YAP (Fig. 8a). Interestingly, the trend observed here with the 3D microchannels was different from that of the 2.5D microgrooves. A minimal ratio of 1.1 lied at ADC5 followed by C10 while a maximum of 1.4 was found in the C20 channels followed by the flat ctrl and then C30 (Fig. 8b). As for the length of the processes of the cells, it increased from D1 to D3 (Fig. 8c). This was not the case for the ctrl due to the confluence of the cells. We also observed a maximum of 65 μm at C20 and a minimum of 12 μm on the flat ctrl. Almost no processes were observed for cells in ADC5 channels. Overall, both for the 2.5D microgrooves and the 3D microchannels, we observed that the YAP N/C ratio is inversely proportional to the total average length of processes per cell, which may be attributed to the degree of differentiation of the cells since it was shown that the lower the YAP N/C ratio, the further the cells are down the neuronal differentiation path [34,35]. An investigation of MRTFA N/C ratio showed that it was mostly at 1 and slightly below. The minimum was found at ADC5 and the maximum at C30 (Fig. 8d and Fig. S6 in the supporting information) which is contrary to the results of SH-SY5Y cells in 2.5D microgrooves. It is difficult to discern the reason behind this behaviour of MRTFA. As for MAP2 average intensity, it followed a similar trend of that of YAP N/C ratio with the exception that a slight decrease from D1 to D3 was noticed (Fig. 8e). For all three proteins, YAP, MRTFA, and MAP2, the trends showed a minimum at ADC5 which may suggest that the increase in cell polarity and 3D confinement has an adverse effect on tension in the cytoskeleton and hence the activation of these mechanotransductive pathways. The difference of results between the 2.5D microgrooves and 3D microchannels alludes to the fact that a 3D full confinement may have a different effect than a 2.5D partial one. This may be related to a number of reasons. First, differentiated cells cultured in C10, ADC10, and ADC5 3D microchannels exhibited a higher aspect ratio and polarity of nuclei compared to those cultured in G10 and G5 2.5D microgrooves. Second, a closed 3D microchannel creates a different microenvironment compared to 2.5D open grooves since nutrition is certainly more dependent on diffusion within the 3D microchannels due to the hindrance that the closed channel represents for the molecules of nutrients to flow to the cells through the medium. Naturally, this problem is expected to have been exacerbated for channels with smaller diameters (i.e. C10, ADC10, and ADC5). Finally, the density of cells in 3D microchannels was much lower than that in 2.5D microgrooves and cell density is known to have an effect on YAP N/C ratio [27]. The apparent difference in the behaviour of cells in 2.5D vs. 3D microenvironments has not been specifically investigated in literature and its exploitation may lead to furthering the understanding of fundamental behaviours of mechanotransductive proteins.

**Fig. 9:**
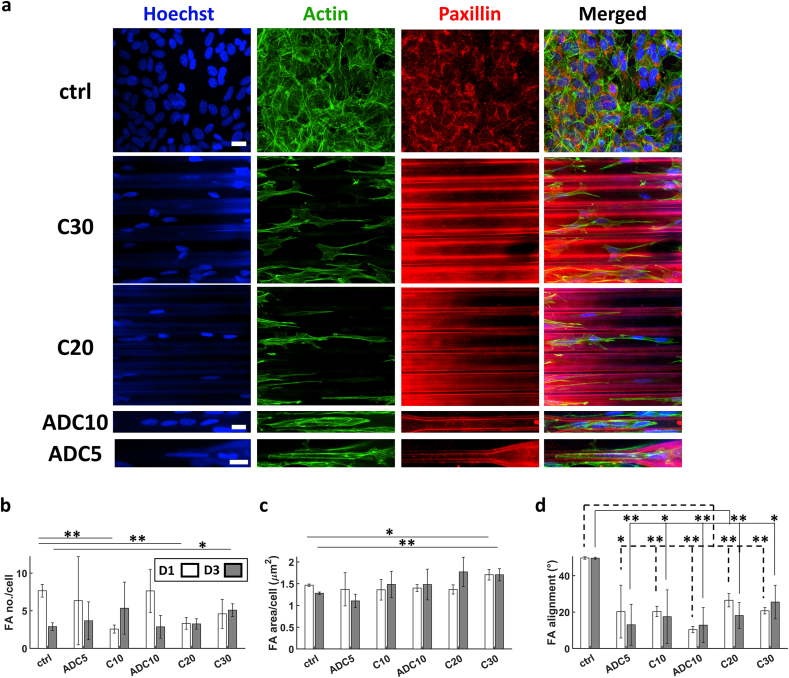
(a) Confocal maximum Z-projection of SH-SY5Y immature neuron-like cells at D3. Scale bars in the first, fourth and fifth rows = 20, 15 and 15 μm respectively. (b) The average number of FAs per cell. (c) Average FA area per cell. (d) Average alignment of FAs. The white and grey bars represent D1 and D3 respectively. ∗ corresponds to a p-value <0.05 and ∗∗ corresponds to a p-value <0.01. P-values were obtained by two-tailed student's t-test. n = 3 samples for each time point.

The terminal ends of the cells interacted with the hatch lines of the 3D microchannels to form FAs as depicted in Fig. 9a. Cells in ADC5 had very few FAs compared to all other 3D microchannels. The average FA number per cell at D3 showed a maximum at C10 and a minimum on the flat ITO-coated glass ctrl with no clear trend (Fig. 9b). On the other hand, the average FA area per cell had a maximum at C20 and a minimum at ADC5 (Fig. 9c) while the alignment of FAs increased (Fig. 9d) with the decrease of the diameter of the 3D microchannels proving the effect of the structures and especially the fine features of the hatch lines on the configuration of the FAs.

### 3.4 The effect of confinement in 2.5D structures on differentiated hiPSC-derived neurons

**Fig. 10:**
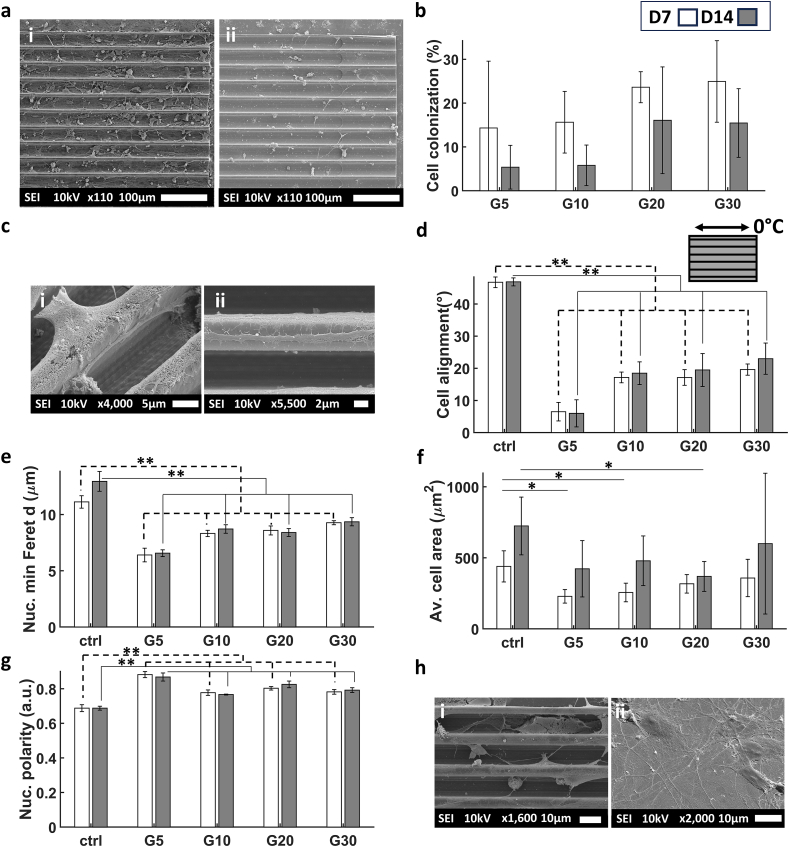
(a) Representative SEM images of hiPSC-neurons (i) at D7 in G20 and (ii) at D14 in G20. (b) The percentage of cell colonization of hiPSC-derived neurons in the 2.5D microgrooves calculated as the ratio between the total area of cell bodies with respect to the total area of the microgrooves. (c) SEM images showing the alignment and connectivity of cells at D7 in (i) G10 and (ii) G5. (d) Degree of cell alignment in the 2.5D microgrooves. (e) The average minimum Feret diameter of the cells. (f) The average area of the cell bodies. (g) The average nuclear polarity (where 0 signifies a perfect circle and 1 a straight line). The white and grey bars represent D7 and D14 respectively. (h) SEM images illustrating the polarity of the cells at D7 in (i) G10 vs. (ii) the flat substrate (ctrl). The white and grey bars represent D7 and D14 respectively. ∗ corresponds to a p-value <0.05 and ∗∗ corresponds to a p-value <0.01. P-values were obtained by two-tailed student's t-test. n = 3 samples for each time point.

Following the investigation conducted on SH-SY5Y differentiated cells in 2.5D microgrooves, we performed a twin study in presence of hiPSC-derived cortical neurons due to their higher physiological relevance in the field of neuro-mechanobiology and *in vitro* disease modelling [[69], [70], [71]]. 2.5D microgrooves of the same size range were employed and hiPSCs were differentiated into immature cortical neurons for 7 and 14 days (i.e. D7 and D14). Cells colonized all 2.5D microgrooves and formed complex networks (Fig. 10a and Fig. S12 in the supporting information), but interestingly, the degree of colonization decreased from D7 to D14 (Fig. 10b) which may indicate that upon further maturation into neurons, these cells prefer to form unaligned clump-like networks (data not shown). As for alignment, qualitative investigation of the cells via SEM imaging showed a high degree of interaction between the cells and the microstructures. The processes of the cells aligned along the lines of the hatch lines and in some cases their filopodia probed in a parallel direction to the lines. The cells also aligned on top of the ridges and showed a high degree of connectivity (Fig. 10c). The variation of the angles of the nuclei with respect to the direction of the 2.5D microgrooves decreased for the smaller grooves as expected (Fig. 10d). The minimum Feret diameters of the nuclei decreased with the decrease of the size of the 2.5D microgrooves as well (Fig. 10e) while the cell area showed a maximum on the flat ctrl substrate and a minimum in the G20 microgrooves (Fig. 10f). In combination with the minimum Feret diameter, the maximum Feret diameter of the nuclei illustrated the high polarity of the cells in all 2.5D microgrooves compared to the ctrl (Fig. 10g and h). Fig. S13 in the supporting information depicts 3D renders of cells in the microchannels.

**Fig. 11:**
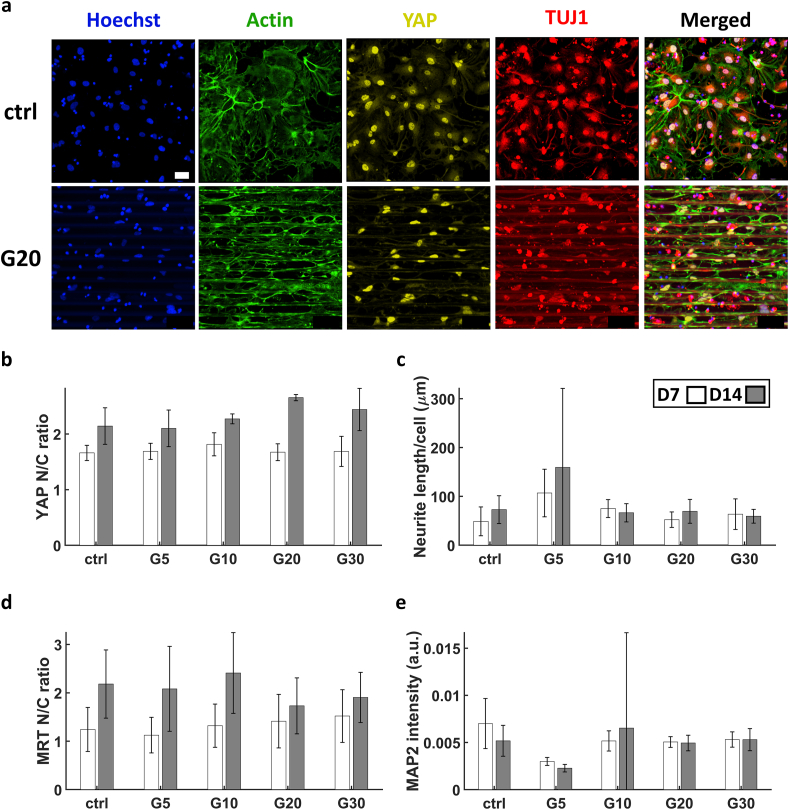
(a) Maximum Z-projection images obtained via confocal microscopy of the hiPSC-neurons at D7 of differentiation. (b) YAP N/C ratio. (c) Average total length of processes per cell. (d) MRTFA N/C ratio. (e) MAP2 average intensity. The white and grey bars represent D7 and D14 respectively. For YAP N/C ratio, n = 3 samples for each time point. For neurite average length, n = 4 samples for each time point. For MRTFA N/C ratio and MAP2 intensity, n = 1 sample for each time point. Scale bar = 50 μm.

As for the YAP N/C ratio, similar to the SH-SY5Y cells, the ratio was always larger than 1 (Fig. 11a and b). There was an increase from D7 to D14 for all cases. At D14, the ratio was larger than 2. A minimum of 2.1 was at G5 and a maximum of 2.7 was at G20 (Fig. 11b). In relation to the cell body area, there seems to be an inversely proportional relationship of YAP N/C ratio. These results are similar to those reported for SH-SY5Y cells therefore the same argumentation for the possible reasons behind this correlation follows. A study of the average total neurite length per cell exhibited a minimum at G30 and a maximum at G5, although for the latter one a relatively high standard deviation was observed (Fig. 11c). However, no noticeable trend was observed nor could we deduce a relationship between this elongation and the YAP N/C ratio. For MRTFA N/C ratio, the maximum was 2.3 at G10 and a minimum of 1.6 was at G20 (Fig. 11d and Fig. S7 in the supporting information). Similar to SH-SY5Y cells in 2.5D microgrooves, differentiated hiPSC-derived neurons at D14 showed more MRTFA N/C ratio albeit with a much larger difference between the two time points in the case of hiPSCs. Having a maximum at G10 for MRTFA N/C ratio in hiPSCs illustrates that the relationship drawn between confinement and MRTFA due to increased mechanical stress can be carried over from SH-SY5Y cells to this more physiologically relevant model of hiPSCs. Since the study performed on hiPSCs here was a proof of principle, we would recommend further investigation into this particular phenomenon in the future with more samples to obtain statistically significant data. The mean intensity of MAP2 showed a maximum at G10 and a minimum at G5 with no clear trend relatable to YAP or FAs (Fig. 11e). This can be seen as contrary to the study of Ankam et al. where they showed that the expression of MAP2 of hESC-derived neurons increased when cultured on nanogratings of 250 nm width and height compared to flat PDMS substrates. This was accompanied by significant alignment of the cells as well [72].However, since MAP2 is an indicator of neuronal maturation, these results may suggest that confinement aids the differentiation of hiPSCs into neurons until a certain extent after which, confinement exhibits an adverse effect on neuronal maturation (i.e. in G5).

**Fig. 12:**
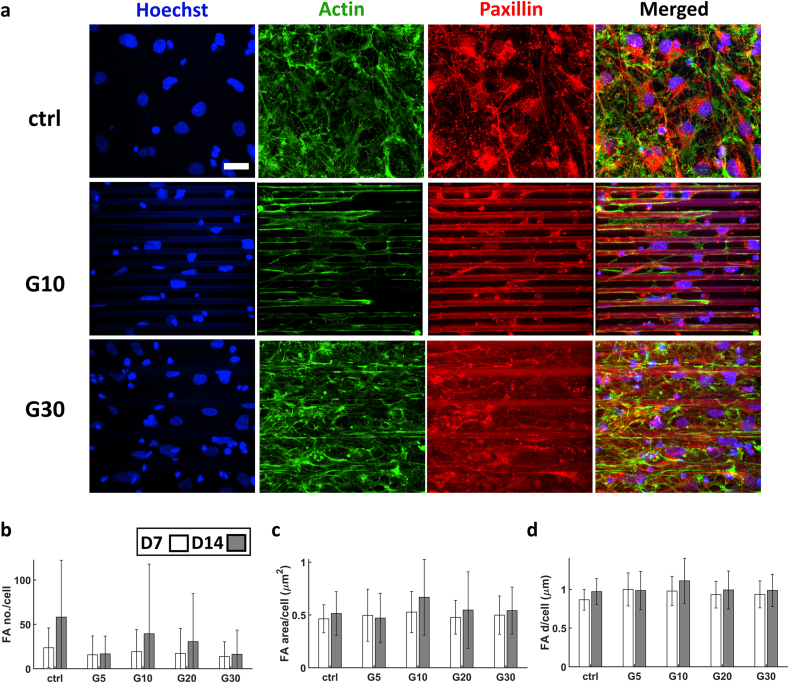
(a) Confocal maximum Z-projection of hiPSC-derived neurons at D7. (b) The average number of FAs per cell. (c) Average FA area per cell. (d) Average FA diameter per cell. The white and grey bars represent D7 and D14 respectively. n = 1 sample for each time point. Scale bar = 30 μm.

The average FA number per cell showed a substantial increase compared to the SH-SY5Y cells (Fig. 12a). This increase in number of FAs was accompanied by a significant decrease in the sizes of FAs for hiPSC-derived neurons (Fig. 12b–d). This is most probably due to the nature of FAs in hiPSC-derived neurons for which we observed how neuronal processes (supported by actin filaments) are much longer, much more branched and with finer protrusions than SH-SY5Y-derived neuron-like cells. In our observations, this lead to the formation of much finer and much more spread FAs in the case of hiPSCs. The maximum of the size of FAs was at G10, but no apparent relationship could be drawn between it and the N/C ratio of YAP, which suggests that the conformation of the cytoskeleton and the area of the cell body are the main effectors on the behaviour of YAP in hiPSC-derived cortical neurons.

## 4 Conclusions

Mechanotransductive proteins such as YAP and MRTFA play a vital role in neuronal development. They are in a constant dynamic state where they are affected by the conformation and polymerization of the actin filaments of the cytoskeleton. This is closely related to the formation and alignment of FAs on different substrates. One of the most interesting mechanical cues that has the ability to alter the conformation of the cytoskeleton is mechanical confinement. In this study, we investigated the effect of confinement, in 2.5D microgrooves and 3D microchannels with diameters ranging from 5 to 30 μm, on YAP, MRTFA N/C ratio and neurite elongation. We cultured SH-SY5Y cells on the microstructures and differentiated them into immature neuron-like cells for 1 and 3 days. Our results showed that, in presence of 2.5D microgrooves, an increase in YAP N/C ratio was accompanied by a decrease in average neurite length. In the 2.5D microgrooves, maximum values of YAP and MRTFA N/C ratios were noticed in the 10 μm grooves at which a decrease was observed and can be tentatively attributed to the increased polarity of the cell or the nucleus in the 5 μm grooves. For MRTFA specifically, it seemed that the increased mechanical stress, and consequently F-actin formation, induced by the smaller grooves, increased its N/C ratio as expected. In the case of the 3D microchannels, the novel alternative designs of the 5 and 10 μm tapered channels substantially fostered cell colonization. YAP and MRTFA N/C ratios had similar trends for which the minimum was found at the 5 μm and the maximum at the 30 μm 3D microchannels. The trend was therefore different from the one observed in the 2.5D microgrooves. This depends on the effects of 3D microarchitecture on the actin in the cytoskeleton compared to 2.5D microgrooves, although further research must be carried out to confirm differences between these microenvironments. Focal adhesions showed a high degree of alignment for all microstructures. We also cultured hiPSCs on the 2.5D microgrooves and differentiated them into immature neurons for 7 and 14 days since this is a more physiologically relevant cell model compared to SH-SY5Y cells. Interestingly, the behaviour of hiPSC-derived cortical neurons was similar to SH-SY5Y cells since the maximum of YAP and MRTFA N/C ratios were observed at the 20 and 10 μm 2.5D microgrooves respectively. No evident relationship was observed between the proteins and the length of neurites. On average, the number of FAs per cell was substantially higher compared to SH-SY5Y cells and their sizes were much smaller. In summary, our results suggest a relationship between YAP and MRTFA in relation to mechanical confinement in 2.5D and 3D engineered microenvironments. A relationship between these proteins and neurite outgrowth could be drawn for SH-SY5Y cells. Most interestingly, the results were different from 2.5D and 3D microenvironments alluding to the fact that mechanical confinement is highly sensitive to geometrical changes in the microenvironment. Further, another reason for the difference between the results is that the supply of nutrients and oxygen can be different in 3D microchannels compared to 2.5D microgrooves. Therefore we suggest in future studies to investigate the expression of hypoxia indicators such as HIF-1α [73] in order to confirm this hypothesis. Future studies attempting to discover additional relationships between these types of mechanical confinement and neuro-mechanobiology could include: the investigation of nuclear mechanotransductive proteins such as Lamin A/C [44] and be extended to disease models (e.g. Alzheimer's disease [31,74]); and the fabrication of confining microarchitectures made of softer biomaterials (e.g. photocrosslinkable hydrogels [75] and elastomers [76] with a Young's modulus in the kPa-MPa range).

## CRediT authorship contribution statement

**Ahmed Sharaf:** Writing – review & editing, Writing – original draft, Visualization, Validation, Software, Methodology, Investigation, Formal analysis, Data curation, Conceptualization. **Jean-Philippe Frimat:** Writing – original draft, Resources, Methodology, Investigation. **Angelo Accardo:** Writing – review & editing, Supervision, Resources, Project administration, Methodology, Investigation, Funding acquisition, Conceptualization.

## Declaration of competing interest

The authors declare that they have no known competing financial interests or personal relationships that could have appeared to influence the work reported in this paper.

## Data Availability

Data will be made available on request.
