# Supplementary material for: Mechanical confinement matters: Unveiling the effect of two-photon polymerized 2.5D and 3D microarchitectures on neuronal YAP expression and neurite outgrowth
Source: Mater Today Bio. 2024 Nov 2;29:101325. doi: 10.1016/j.mtbio.2024.101325 (PMC11576396; doi:10.1016/j.mtbio.2024.101325)
Supplement: Multimedia component 1 [file mmc1.pdf]

Mechanical confinement matters: unveiling the effect of two-photon polymerized 2.5D and 3D microarchitectures on neuronal YAP expression and neurite outgrowth

Supporting Information

Ahmed Sharaf[a,*], Jean-Philippe Frimat[b], Angelo Accardo[a,*]

[a]Department of Precision and Microsystems Engineering, Faculty of Mechanical Engineering, Delft University of Technology, Mekelweg 2, 2628 CD Delft, the Netherlands
[b]Department of Human Genetics, Leiden University Medical Center, 2333 ZA Leiden, the Netherlands

*Corresponding authors: a.m.s.e.sharaf@tudelft.nl, a.accardo@tudelft.nl

CAD renderings and dimensions of all microstructures.

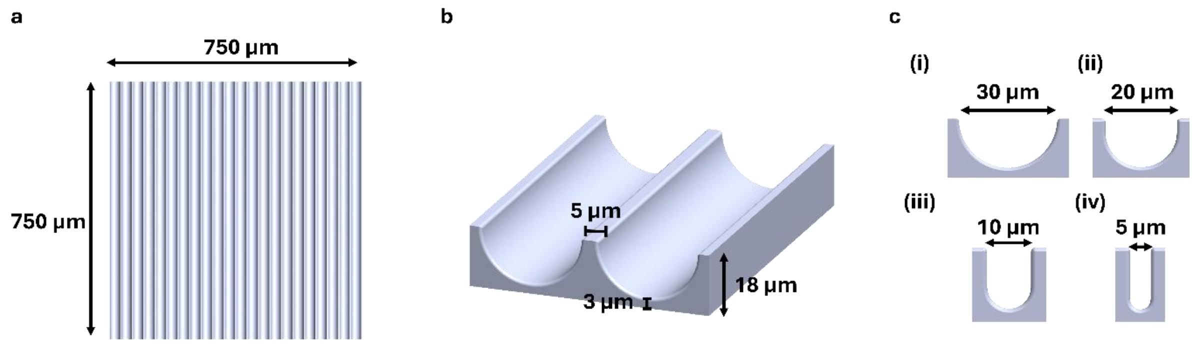

Fig. S1. CAD renderings of the 2.5D microgrooves created by SOLIDWORKS. (a) Top view and dimensions of a representative array. (b) Isometric zoomed in view and detailed dimensions of the microgrooves. (c) Front view and diameters of each array.

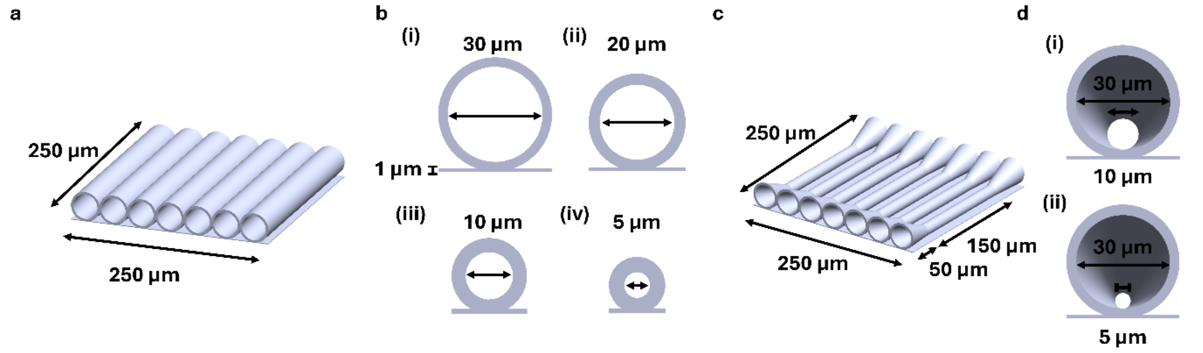

Fig. S2. CAD renderings of the 3D microchannels created by SOLIDWORKS. (a) Isometric view and dimensions of one C30 array of microchannels. (b) Front view and diameters of (i) C30, (ii) C20, (iii) C10, and (iv) C5. (c) Isometric view and dimensions of one ADC10 array of microchannels. (d) Front view and dimensions of (i) ADC10 and (ii) ADC5.

Elaboration of analysis pipelines.

Fig. S3 shows the pipeline for identifying cell bodies and neurites (processes). After obtaining maximum z-projection images by Fiji (Fig. S3a), Cellpose was employed to identify nuclei via machine learning (Fig. S3b). Consequently, the identified nuclei were imported into CellProfiler as primary objects (Fig. S3c) to be used as seeds for identifying the bodies of the cells by using the F-actin staining (Fig. S3d). Cell bodies were identified by using an Otsu thresholding method. Afterwards, identified cell bodies were turned into objects (Fig. S3e) to perform further analysis such as calculating cell area. Nuclei were also used as seeds to identify processes of the cells (Fig. S3f). For this step, nuclei had to be dilated in order to account for the cell body area that must be excluded from the calculation of the length of the processes.

Focal adhesions (FAs) were identified as shown in Fig. S4. First, the cell bodies were identified as already mentioned (Fig. S4a,b). FAs were then identified in CellProfiler as primary objects (Fig. S4c) based on the paxillin channel (Fig. S4d). The Robust Background thresholding method was employed to identify FAs. Finally, FAs were related to the relevant cell body as shown in Fig. S4e.

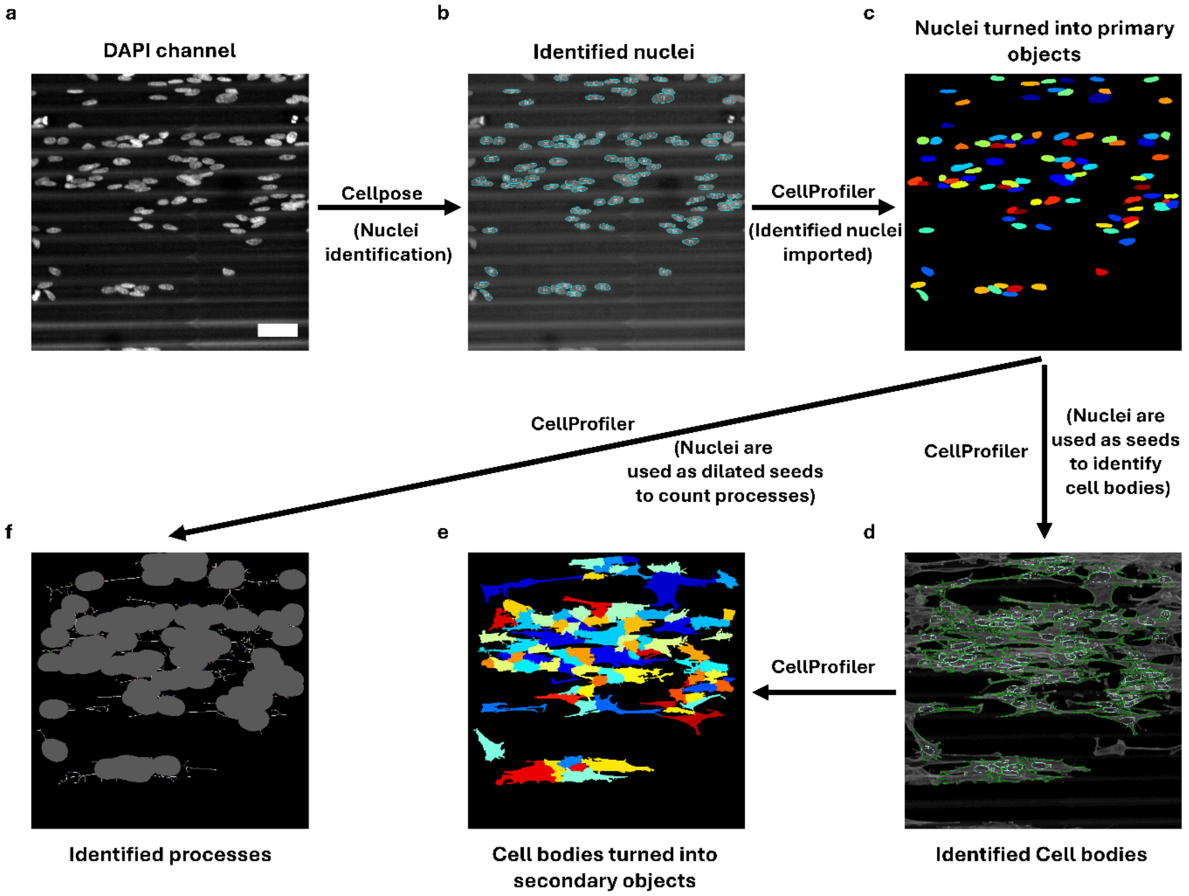

Fig. S3. Analysis pipeline for identification of cell bodies and neurites (processes). (a) Representative maximum z-projection of a DAPI channel of C20 (created by using Fiji). (b) Identified nuclei via Cellpose machine learning-based plugin are indicated by the blue contours. (c) Identified nuclei were imported into CellProfiler as primary objects. (d) Identified cell bodies shown as green contours and overlayed on the actin channel. (e) Identified cell bodies were transformed into objects in CellProfiler for further analysis. (f) Neurites (processes) of cells (as indicated by the white branched lines) were identified by using nuclei as seeds after their dilation. Scale bar = 50 µm.

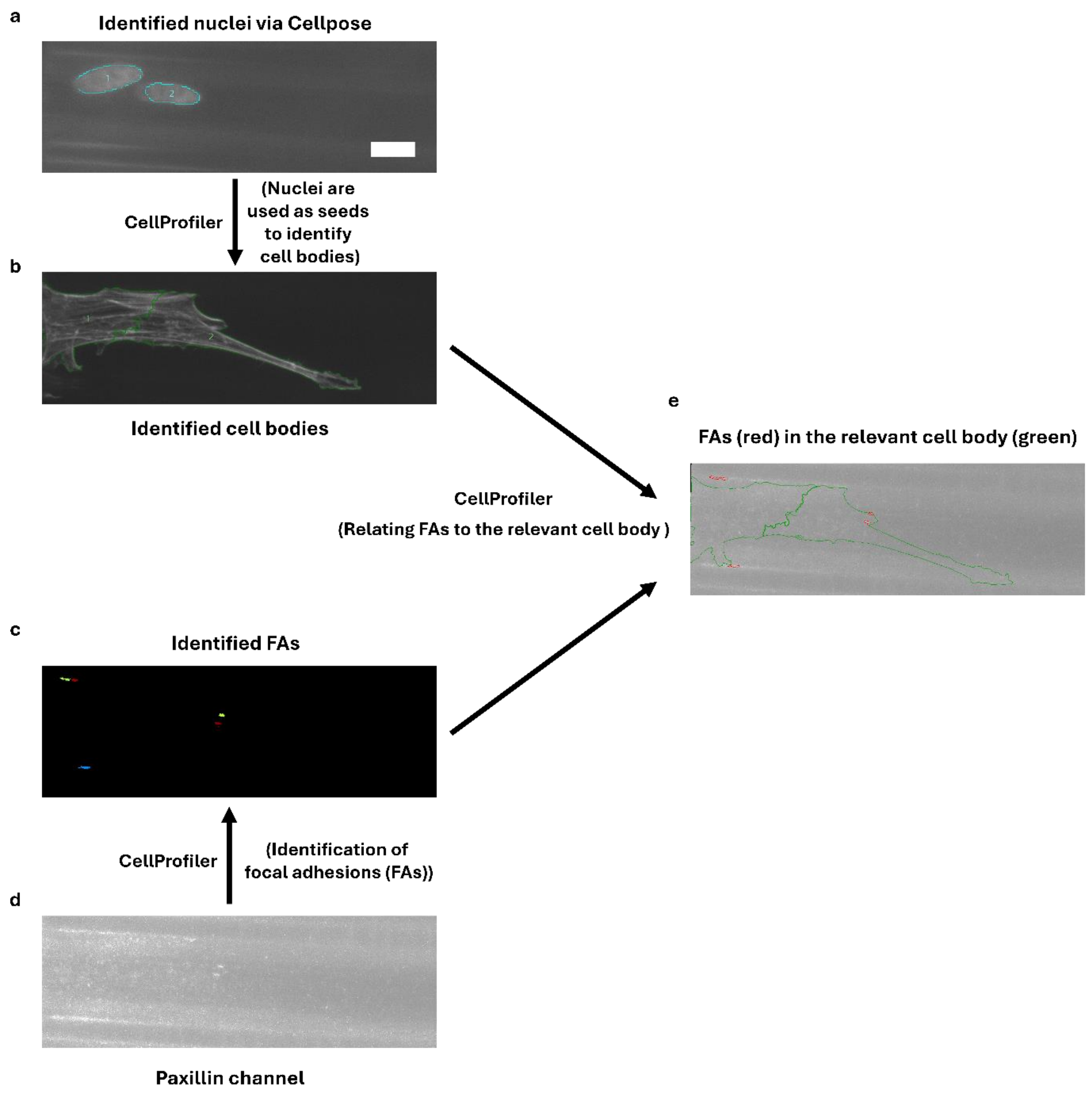

Fig. S4. Analysis pipeline for identification of focal adhesions (FAs). (a) Identified nuclei via Cellpose as mentioned earlier. (b) Identified nuclei were imported into CellProfiler and used as seeds to identify cell bodies as mentioned earlier. (c) FAs were identified in CellProfiler by using the paxillin channel (d). (e) identified FAs (red contours) were related to the relevant cell body (green contours) in CellProfiler for further analysis. Scale bar = 10 µm.

Fluorescence images of MRTFA.

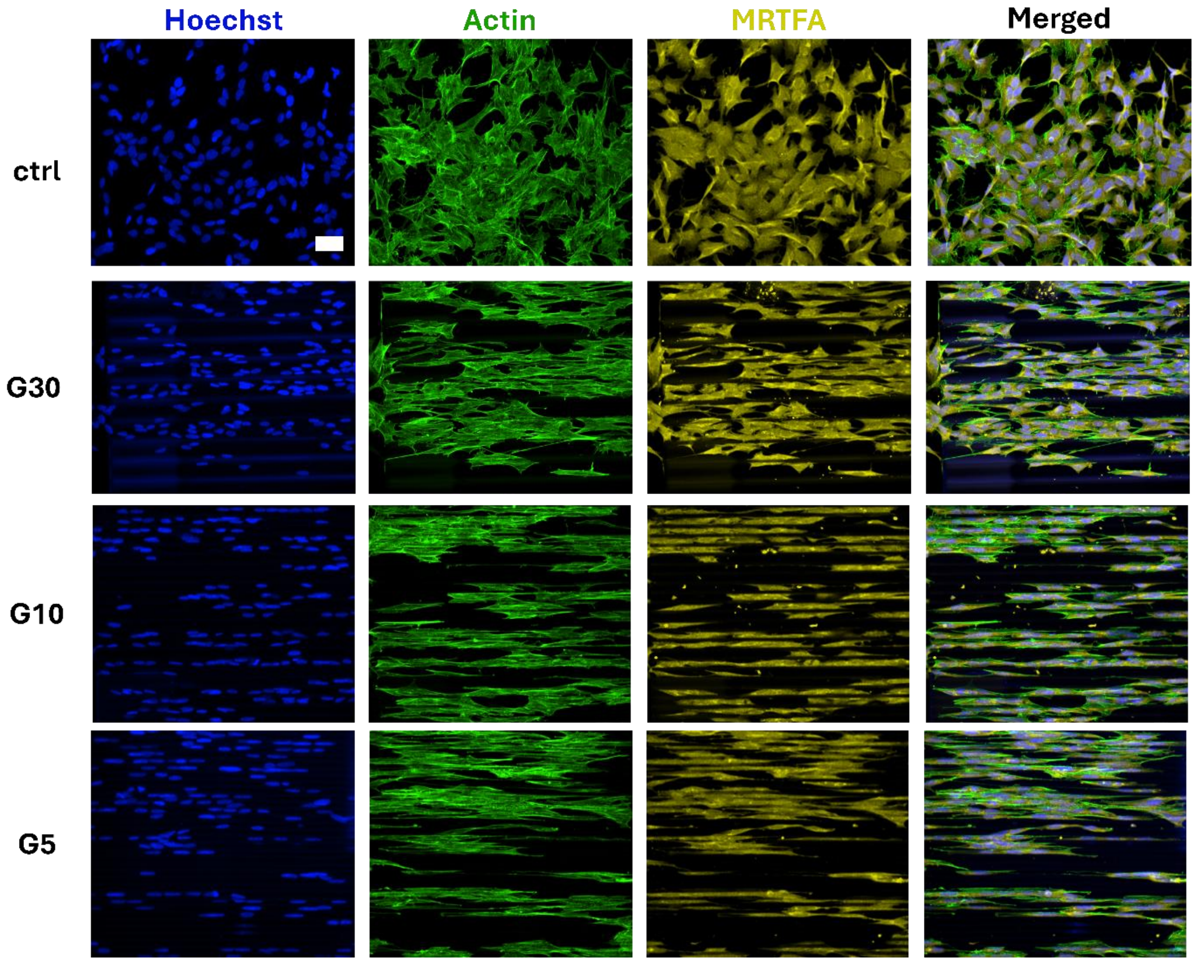

Fig. S5. Maximum Z-projection images obtained via confocal microscopy of the SH-SY5Y cells at D3 of differentiation on ctrl, G30, G10 and G5 showcasing the expression of MRTFA in the cells. Scale bar = 50 µm.

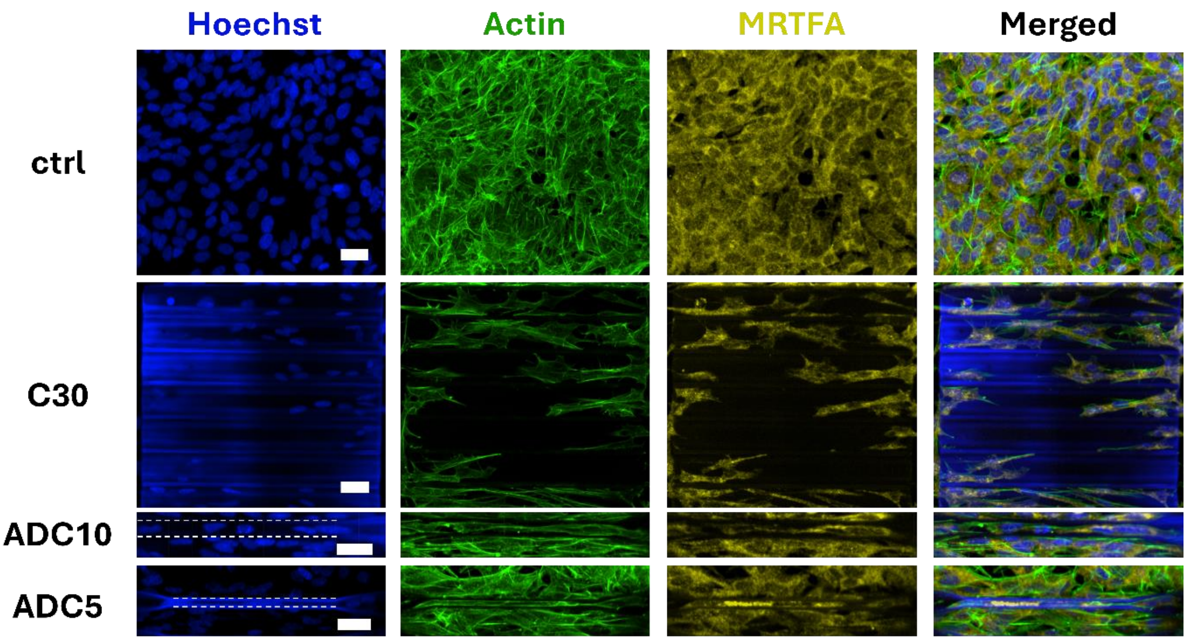

Fig. S6. Maximum Z-projection images obtained via confocal microscopy of the SH-SY5Y cells at D3 of differentiation on ctrl, C30, ADC10 and ADC5 showcasing the expression of MRTFA in the cells. The dashed white lines in ADC10 and ADC5 represent the channel walls. Scale bar = 30 µm.

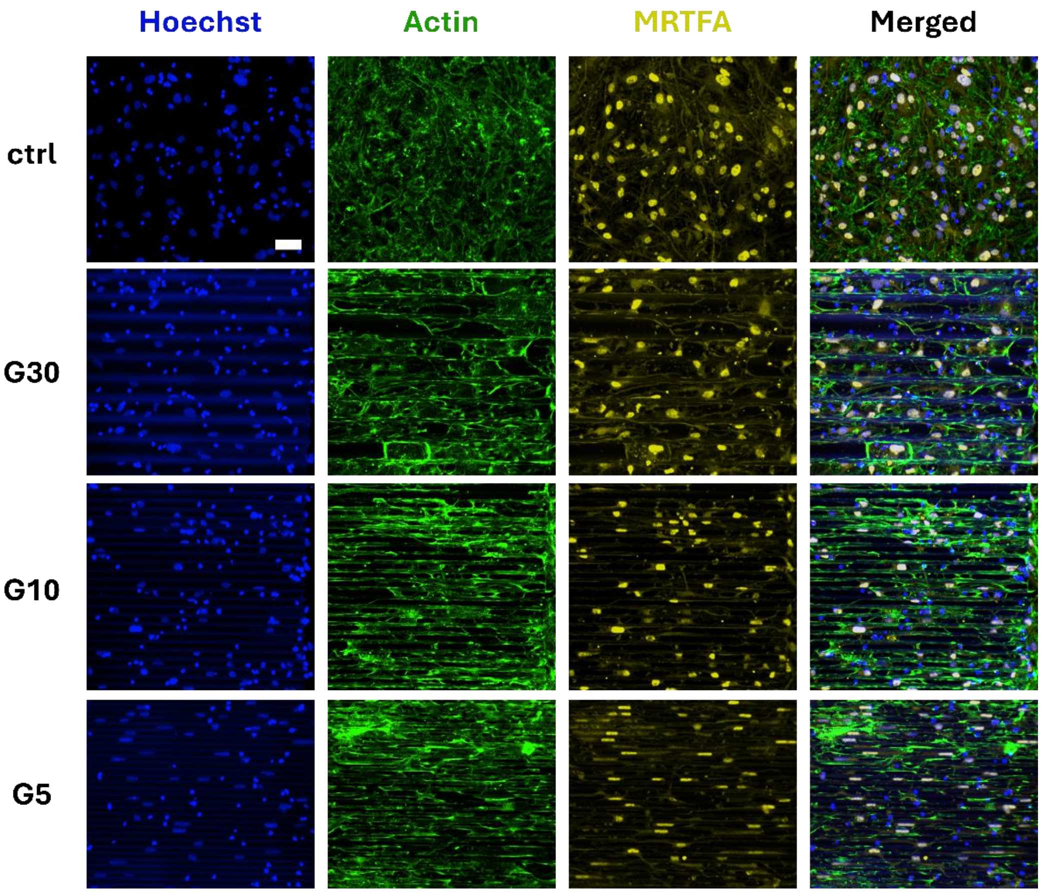

Fig. S7. Maximum Z-projection images obtained via confocal microscopy of the hiPSC cells at D14 of differentiation on ctrl, G30, G10 and G5 showcasing the expression of MRTFA in the cells. Scale bar = 50 µm.

Channels length determination.

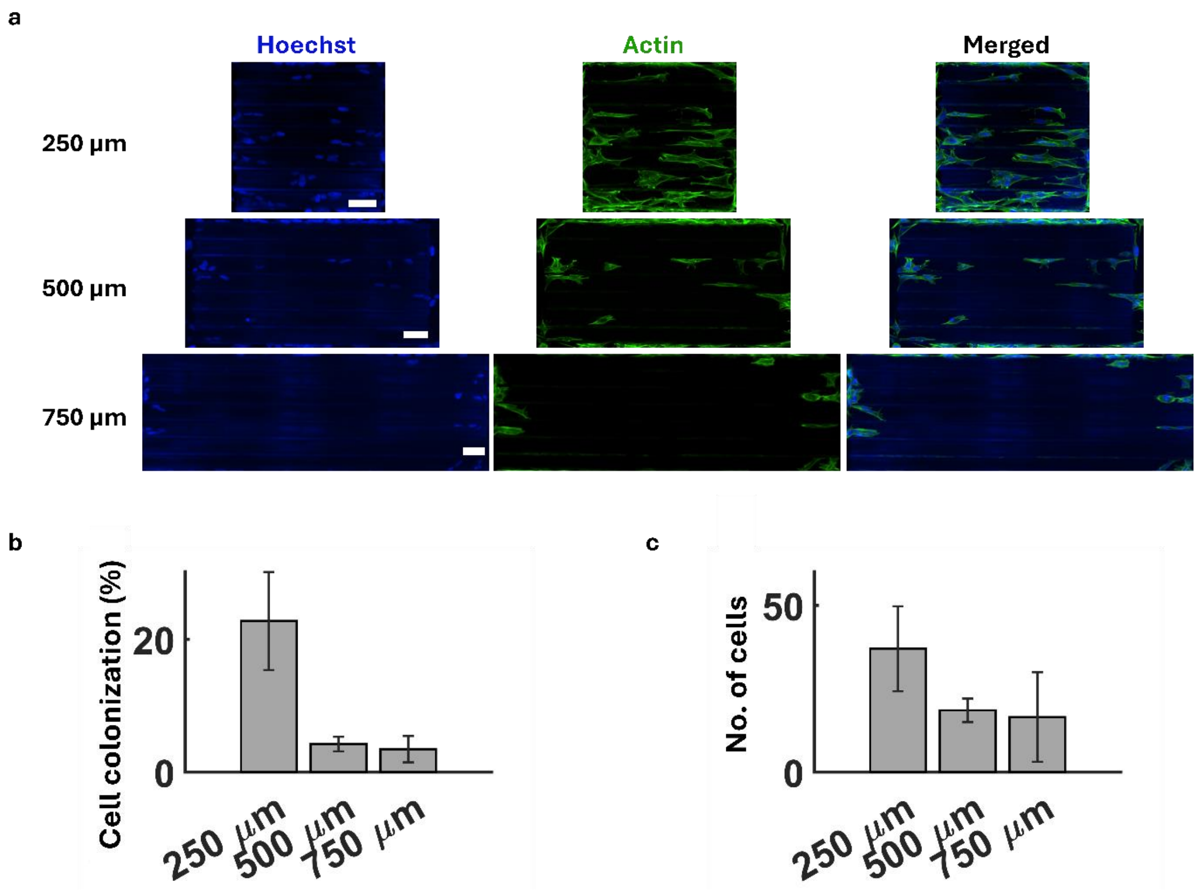

Fig. S8. (a) Maximum Z-projection images obtained via confocal microscopy of the SH-SY5Y cells at D1 of differentiation in channels of 30 µm diameter and lengths of 250, 500, and 750 µm. (b) Percentage of cell colonization calculated as the ratio of the summation of the areas of cell bodies to the area of the arrays of microchannels. (c) The average number of cells per array of channels. Scale bar = 50 µm.

Average area of nuclei in microchannels.

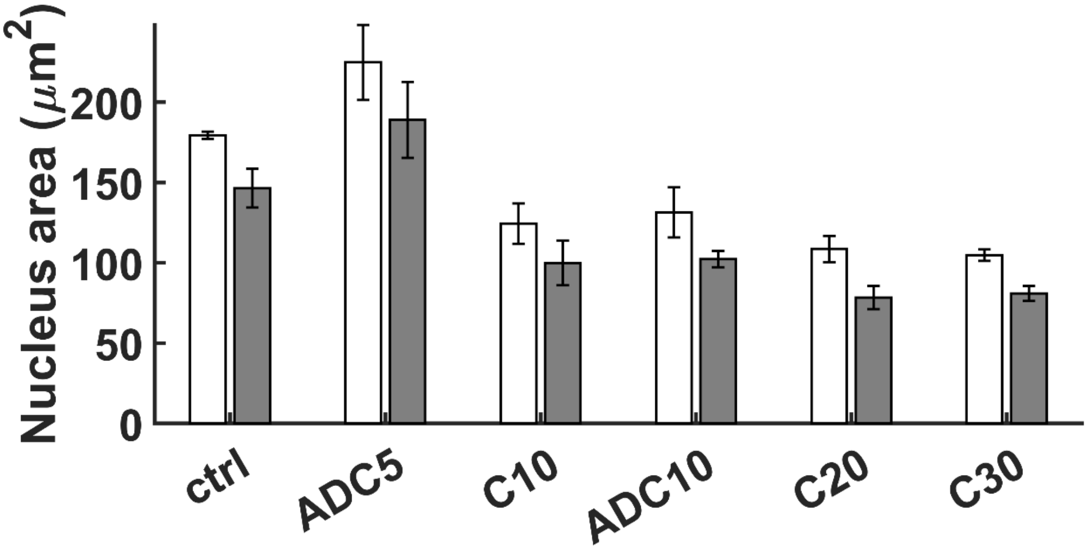

Fig. S9. Average area of nuclei of SH-SY5Y-differentiated cells in the microchannels.

3D renderings of confocal images of SH-SY5Y derived immature neurons.

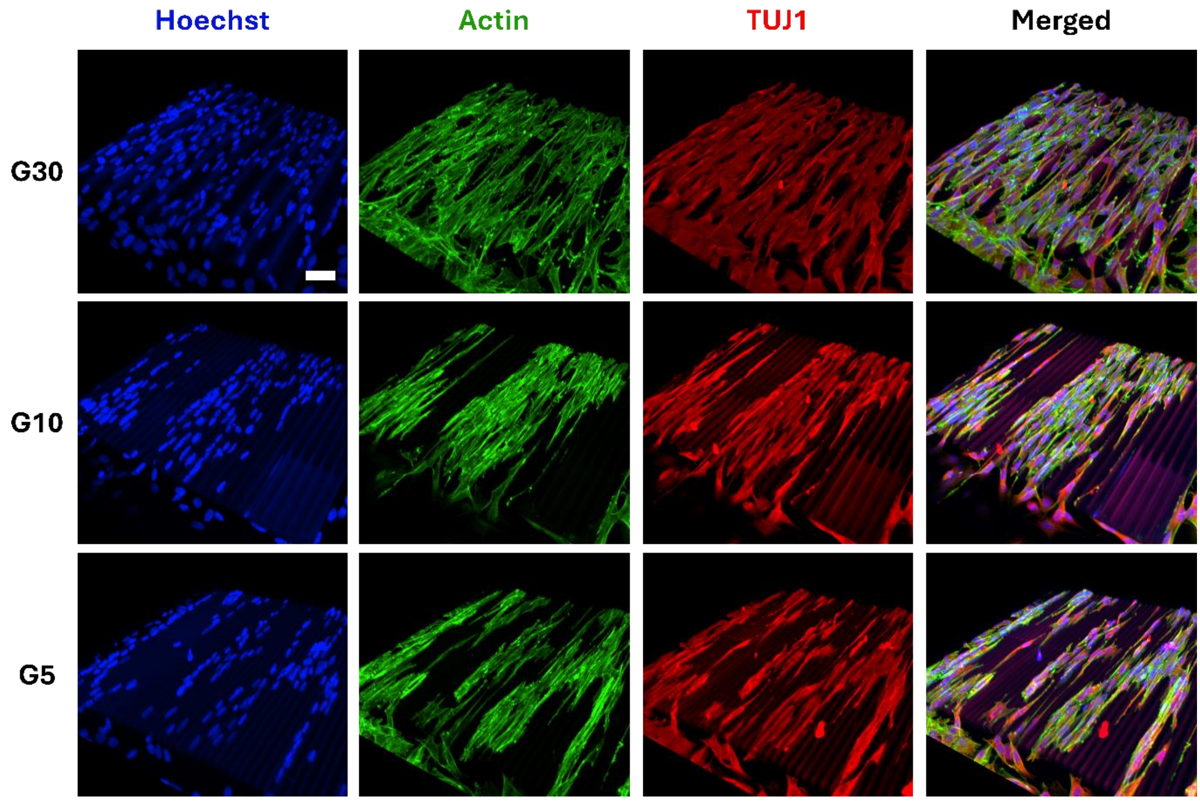

Fig. S10. Representative 3D reconstructions obtained via confocal microscopy of SH-SY5Y cells in microgrooves at D3 of differentiation. Scale bar = 50 µm.

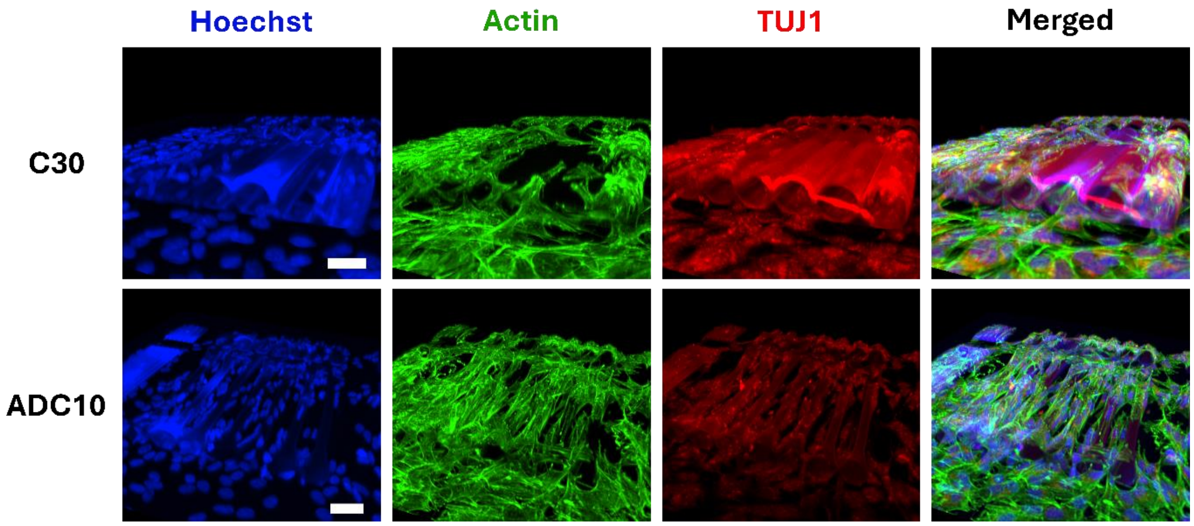

Fig. S11. Representative 3D reconstructions obtained via confocal microscopy of SH-SY5Y cells in microchannels at D3 of differentiation. Scale bar = 50 µm.

Illustration of networks formed between hiPSC derived immature neurons in the microgrooves.

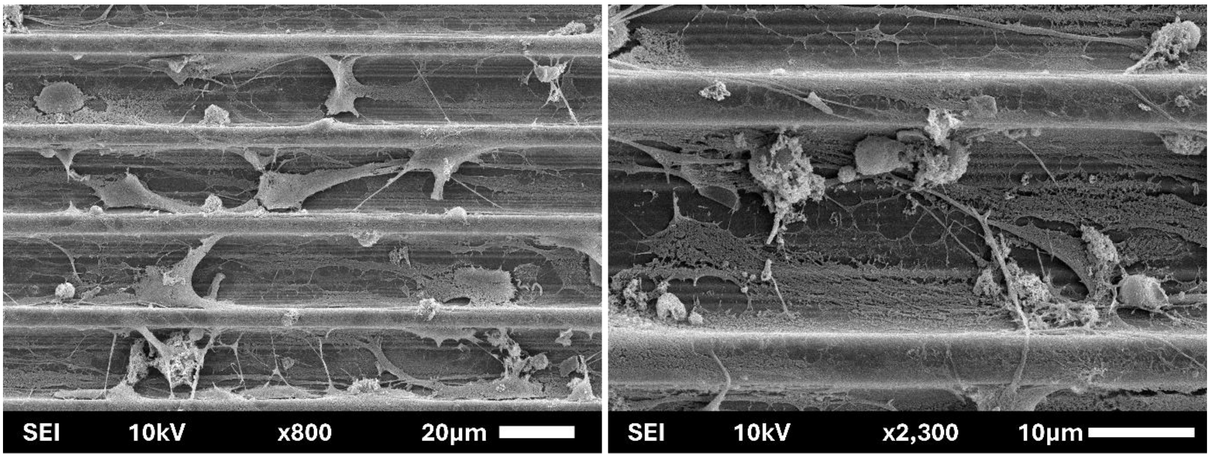

Fig. S12. Representative SEM images of hiPSC derived immature neurons in G20 at D7 illustrating the connectivity and complex networks formed between the cells.

3D renderings of confocal images of hiPSC derived immature neurons.

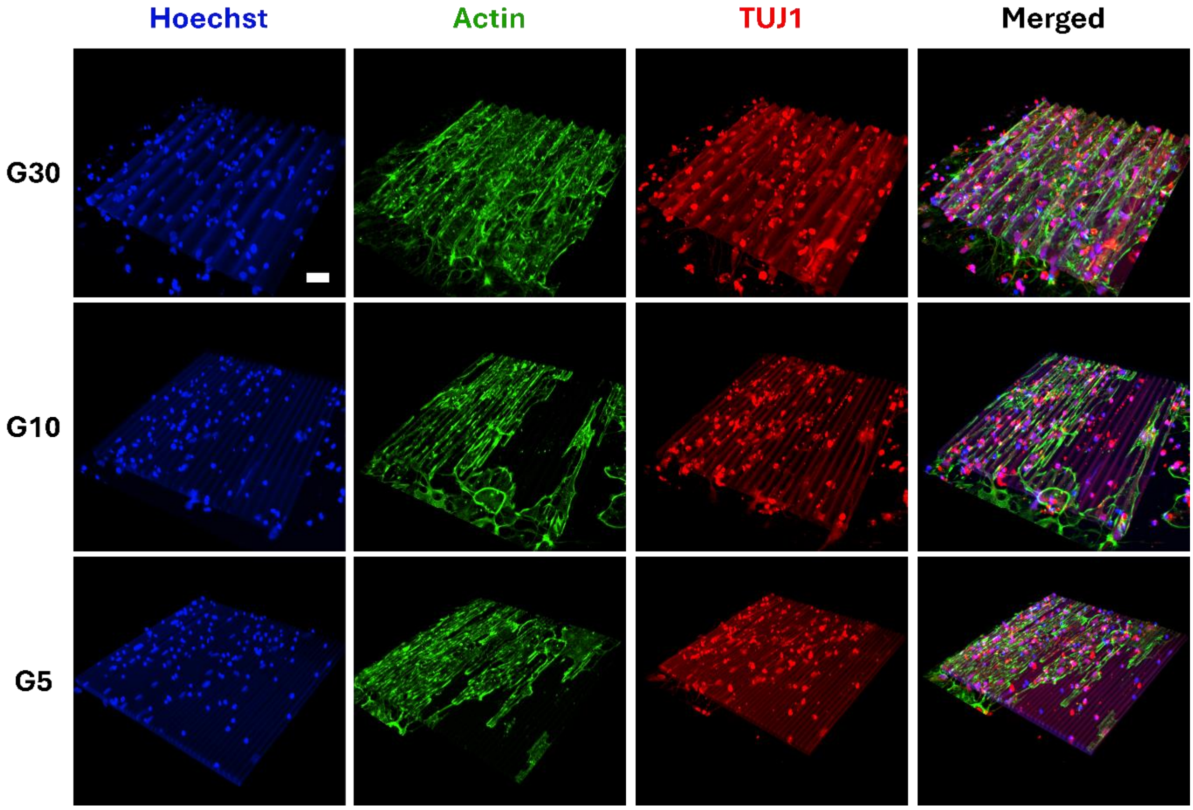

Fig. S13. Representative 3D reconstructions obtained via confocal microscopy of hiPSC cells in microgrooves at D14 of differentiation. Scale bar = 50 µm.
